# Supplementary material for: Exploiting frequent and specific expression of PRL3 in pediatric solid tumors for first-in-child use of PRL3-zumab humanized antibody
Source: Mol Ther Oncolytics. 2023 Aug 18;30:153–66. doi: 10.1016/j.omto.2023.08.006 (PMC10477756; doi:10.1016/j.omto.2023.08.006)
Supplement: Document S1. Figure S1 and Tables S1–S6 [file mmc1.pdf]

OMTO, Volume *30*

Supplemental information

Exploiting frequent and specific expression of PRL3 in pediatric solid tumors for first-in-child use of PRL3-zumab humanized antibody

Amos Hong Pheng Loh, Min Thura, Abhishek Gupta, Sheng Hui Tan, Kelvin Kam Yew Kuan, Koon Hwee Ang, Khurshid Merchant, Kenneth Tou En Chang, Hui Yi Yon, Yong Chen, Mathew Hern Wang Cheng, Arjandas Mahadev, Matthew Chau Hsien Ng, Michaela Su-Fern Seng, Prasad Iyer, Pei Ling Chia, Shui Yen Soh, and Qi Zeng

Table S1. Clinical and pathological features of patients with primary tumors and matched normal tissues

Patient no	Sample ID	Specimen type	Tumor site	Diagnosis	Gender	Age at diagnosis (y)	Extent	PRL3 expr-ession
Neuroblastoma								
1	1T	Primary tumor	Adrenal gland	Nodular ganglioneuroblastoma	Male	3.38	Unknown	+
2	2T	Primary tumor	Retroperitoneum	Differentiating neuroblastoma	Female	3.39	Unknown	+
3	3T	Primary tumor	Retroperitoneum	Differentiating neuroblastoma	Male	2.90	Unknown	+
4	4T	Primary tumor	Retroperitoneum	Intermixed ganglioneuroblastoma	Female	2.67	Metastatic	+
5	5T	Primary tumor	Retroperitoneum	Differentiating neuroblastoma	Male	1.72	Metastatic	+
6	6N	Matched normal	Retroperitoneum	Neuroblastoma	Male	6.22	Unknown	-
7	7T	Primary tumor	Adrenal gland	Neuroblastoma	Male	7.56	Metastatic	+
	7N	Matched normal						-
8	8T	Primary tumor	Liver metastasis	Neuroblastoma	Female	1.74	Metastatic	+
	8N	Matched normal						-
Wilms tumor								
9	1T	Primary tumor	Kidney	Wilms tumor	Male	1.93	Localized	-
10	2T	Primary tumor	Kidney	Wilms tumor	Male	4.28	Localized	-
11	3T	Primary tumor	Kidney	Wilms tumor	Female	0.25	Localized	+
12	4T	Primary tumor	Kidney	Wilms tumor	Female	2.56	Localized	+
	4N	Matched normal						-
13	5T	Primary tumor	Kidney	Wilms tumor	Female	7.22	Localized	-
	5N	Matched normal						-
14	6T	Primary tumor	Kidney	Wilms tumor	Female	2.84	Localized	+
	6N	Matched normal						-
15	7T	Primary tumor	Kidney	Wilms tumor	Female	5.48	Unknown	-
	7N	Matched normal						-
Osteosarcoma								
16	1T	Primary tumor	Tibia	Conventional osteosarcoma	Male	18.35	Localized	-

17	2T	Primary tumor	Femur	Conventional osteosarcoma	Female	12.98	Localized	-
18	3T	Primary tumor	Femur	Conventional osteosarcoma	Male	11.75	Localized	-
19	4T	Primary tumor	Skeletal bone NOS	Conventional osteosarcoma	Male	Unknown	Unknown	+
20	5T	Primary tumor	Lower limb	Conventional osteosarcoma	Female	Unknown	Unknown	+
21	6T	Primary tumor	Lower limb	High grade surface osteosarcoma	Female	Unknown	Unknown	-
22	7M	Metastatic tumor	Lung nodule	Metastatic osteosarcoma	Female	Unknown	Metastatic	+
23	8M	Metastatic tumor	Lung nodule	Metastatic osteosarcoma	Female	Unknown	Metastatic	+
Rhabdomyosarcoma and non-rhabdomyosarcoma soft tissue sarcomas								
24	1T	Primary tumor	Peritoneum	Desmoplastic small round cell tumor	Female	11.16	Regional	+
25	2T	Primary tumor	Lower limb	Alveolar soft part sarcoma	Female	13.22	Regional	+
26	3T	Primary tumor	Chest wall	Undifferentiated sarcoma	Female	13.26	Regional	-
27	4T	Primary tumor	Lower limb	Chondroblastoma	Male	Unknown	Unknown	-
28	5T	Primary tumor	Chest wall	Embryonal rhabdomyosarcoma	Male	3.50	Regional	+
	5N	Matched normal						-
29	6T	Primary tumor	Mandible, parotid	Alveolar rhabdomyosarcoma	Male	1.59	Regional	+
	6N	Matched normal						-
30	7T	Primary tumor	Upper limb and shoulder	Leiomyosarcoma	Male	Unknown	Unknown	+
	7N	Matched normal						-
Renal sarcomas								
31	1T	Primary tumor	Kidney	Rhabdoid tumor of kidney	Male	0.35	Metastatic	+
32	2T	Primary tumor	Kidney	Rhabdoid tumor of kidney	Female	Unknown	Unknown	+
	2N	Matched normal						-
33	3T	Primary tumor	Kidney	Clear cell sarcoma of kidney	Male	Unknown	Unknown	-
	3N	Matched normal						-
34	4T	Primary tumor	Kidney	Clear cell sarcoma of kidney	Male	Unknown	Unknown	+
	4N	Matched normal						-
35	5T	Primary tumor	Kidney	Post-chemotherapy renal sarcoma	Male	Unknown	Unknown	-
	5N	Matched normal						-

Table S2. Clinical and pathological features of patients with primary tumors and matched relapsed tumors

Patient no	Sample ID	Specimen type	Diagnosis	Age at diagnosis (y)	Gender	Extent	Survival status	PRL3 expression
Neuroblastoma								
1	1T	Primary tumor	Differentiating neuroblastoma	6.09	Female	Metastatic	Alive	-
	1R	Relapse tumor		6.39				+
Wilms tumor								
9	1T	Primary tumor	Favorable histology Wilms tumor	5.53	Male	Metastatic	Alive	+
	1R	Relapse tumor		5.91				+
10	2T	Primary tumor	Favorable histology Wilms tumor	4.23	Male	Localized	Dead	+
	2R	Relapse tumor		6.63				+
Osteosarcoma								
16	1T	Primary tumor	High-grade chondroblastic osteosarcoma	8.00	Male	Localized	Dead	+
	1R	Relapse tumor		9.63				-
17	2T	Primary tumor	High-grade conventional osteosarcoma	11.94	Female	Metastatic	Alive	+
	2R	Relapse tumor		14.78				+
18	3T	Primary tumor	High-grade conventional osteosarcoma	8.16	Male	Metastatic	Alive	-
	3R	Relapse tumor		9.14				+
Rhabdomyosarcoma and non-rhabdomyosarcoma soft tissue sarcoma								
24	1T	Primary tumor	Malignant mesothelioma	14.35	Female	Metastatic	Alive	-
	1R	Relapse tumor		14.55				-
25	2T	Primary tumor	PAX7-FKHR translocation positive alveolar rhabdomyosarcoma	2.33	Male	Metastatic	Alive	-
	2R	Relapse tumor*		5.18				+

*Tumor from second relapse; all other relapse tumors listed above are from first relapse episode

Table S3: Treatment history of initial tumor & serial relapses

Type of Treatment	Treatment regimen	Duration
Initial treatment		
1. Chemotherapy	IVADO (ifosfamide, vincristine, actinomycin, doxorubicin) according to EpSSG RMS 2005 metastatic arm	6 months
2. Surgery	Local control resection with regional lymph node sampling	1 day
3. Radiotherapy	Resected primary tumor site and regional nodal basin	1 month
4. Maintenance Chemotherapy	Oral cyclophosphamide and IV vinorelbine	12 months
First relapse (2 months after stopping maintenance therapy)		
1. Salvage chemotherapy	VIT (vincristine, irinotecan, temozolomide)	8 cycles over 6 months
2. Radiotherapy	Whole lung with boost to involved areas	6 weeks
Second relapse (7 months after radiotherapy)		
1. Short bridging chemotherapy	IV vinorelbine and oral cyclophosphamide	10 days
2. Salvage chemotherapy	VTC (vincristine, topotecan, cyclophosphamide)	7 cycles over 5 months
3. Surgery	Local control resection of distant relapse sites	1 day
4. Radiotherapy	Resected distant relapse sites	6 weeks
5. Experimental NK cell therapy	Activated NK cell infusion	1 day
Third relapse (1 month after activated NK cell infusion)		
1. Salvage chemotherapy	IV bevacizumab, gemcitabine, docetaxel	8 cycles over 6 months
2. Surgery	Drainage of persistent malignant effusion	
3. Maintenance therapy	IV temsirolimus and vinblastine	1 month
	IV vinblastine	2 months
Fourth relapse (during maintenance therapy)		1 day
1. Palliative chemotherapy	IV carboplatin and etoposide	8 cycles over 6 months
2. Palliative radiation	Focal to involved distant sites	2 sessions
Fifth relapse (1 month after IV carboplatin & etoposide)		

EpSSG: European pediatric Soft Tissue Sarcoma Study Group; RMS: rhabdomyosarcoma; IV: intravenous; NK: Natural killer cells

Table S4: Salvage strategy treatment plan for 5th relapse

No.	Type of Treatment	Days	Treatment regimen
1	Bridging salvage chemo	-21	IV Vinorelbine dose 1
		-14	IV Vinorelbine dose 2
2	Hypofractionated palliative RT to left chest tumors	-11	Session 1
		-10	Session 2
3	PRL3-zumab compassionate trial	1*	C1D1 4mg/kg in 2 divided doses
		8	C1D8 (4mg/kg)
		18	C2D1 (4.8mg/kg)
4	Hypofractionated palliative RT to left chest tumors	22	Session 3
		23	Session 4
5	PRL3-zumab compassionate trial	28	C2D11 (5.5mg/kg)
	Disease assessment	37	CT chest / Whole Body PET-CT
6	PRL3-zumab compassionate trial	38	C3D1 (5mg/kg)
		48	C3D11 (5mg/kg)

IV: intravenous, CT: computed tomography scan

*C1D1 considered as day 1

Table S5: PRL3-zumab treatment record

Cycle	Day	Body weight	Actual Dose (mg)	PK sample collection
1	1 (C1D1)	14.2 kg	28mg (2mg/kg, AM)<br>28mg (2mg/kg, PM)	pre-C1D1 dose 1 & end of infusion<br>pre-C1D1 dose 2 & end of infusion
	8 (C1D8)	14.3 kg	57mg (4mg/kg)	pre-C1D8 & end of infusion
2	1 (C2D1)	14.7 kg	70mg (4.8 mg/kg)	pre-C2D1 & end of infusion
	11(C2D11)		80mg (5.4mg/kg)	

Table S6: Blood parameters at treatment time points

Parameter	Range	Baseline		Pre-chemo		After RT	Pre-C1D1	Pre-C1D8	Pre-C2D1
		(day -27)	(day -23)	(day -20)	(day -13)	(day -6)	(day 1)*	(day +8)	(day +18)
Full Blood Count									
Haemoglobin	13.5 – 18 g/L	11.2	10.6	9.5	8.5	7	11	11	11.6
WBC count	4.0 – 11.0 x10^9/L	4.4	5.49	4.41	2.02	0.63	7.38	4.73	3.86
Platelet count	140 – 440 x 10^9/L	45	27	32	33	54	48	60	68
RBC count	4.5 – 6.5 x10^9/L	3.82	3.73	3.25	2.9	2.39	3.82	3.71	3.7
Lymphocyte	1.5 – 4 x10^9/L	1.19	1.15	0.68	0.53	some	0.59	0.54	0.62
Neutrophil	2 – 7.5 x 10^9/L	2.68	3.68	2.89	1.37	few	5.46	3.43	2.2
Monocyte	0.2 – 0.8 10^9/L	0.48	0.6	0.82	0.12		1.25	0.69	0.85
Eosinophil	0.04 – 0.4 x 10^9/L	0.04	0.05	0	0		0	0.02	0.15
Basophil	<0.21 x10^9/L	0	0	0.02	0		0	0.05	0.04
Liver Function Test									
Total protein	60-80 g/L	55		55		58	62		
Albumin	40-51 g/L	33	33	35	36	36	40	40	
Total bilirubin	1.7 -20.5 umol/L	8		4		4	6		
Direct bilirubin	<5.1 umol/L	4		<2		<2	3		
ALP	<130 U/L	190		224		208	204		
ALT	<51 U/L	24		31		34	48		
AST	<51 U/L	32		40		39	44		

WBC: white blood cell, RBC: red blood cell, ALP: alkaline phosphatase, ALT: alanine transaminase, AST: aspartate transaminase
*C1D1 considered as day 1

Fig S1

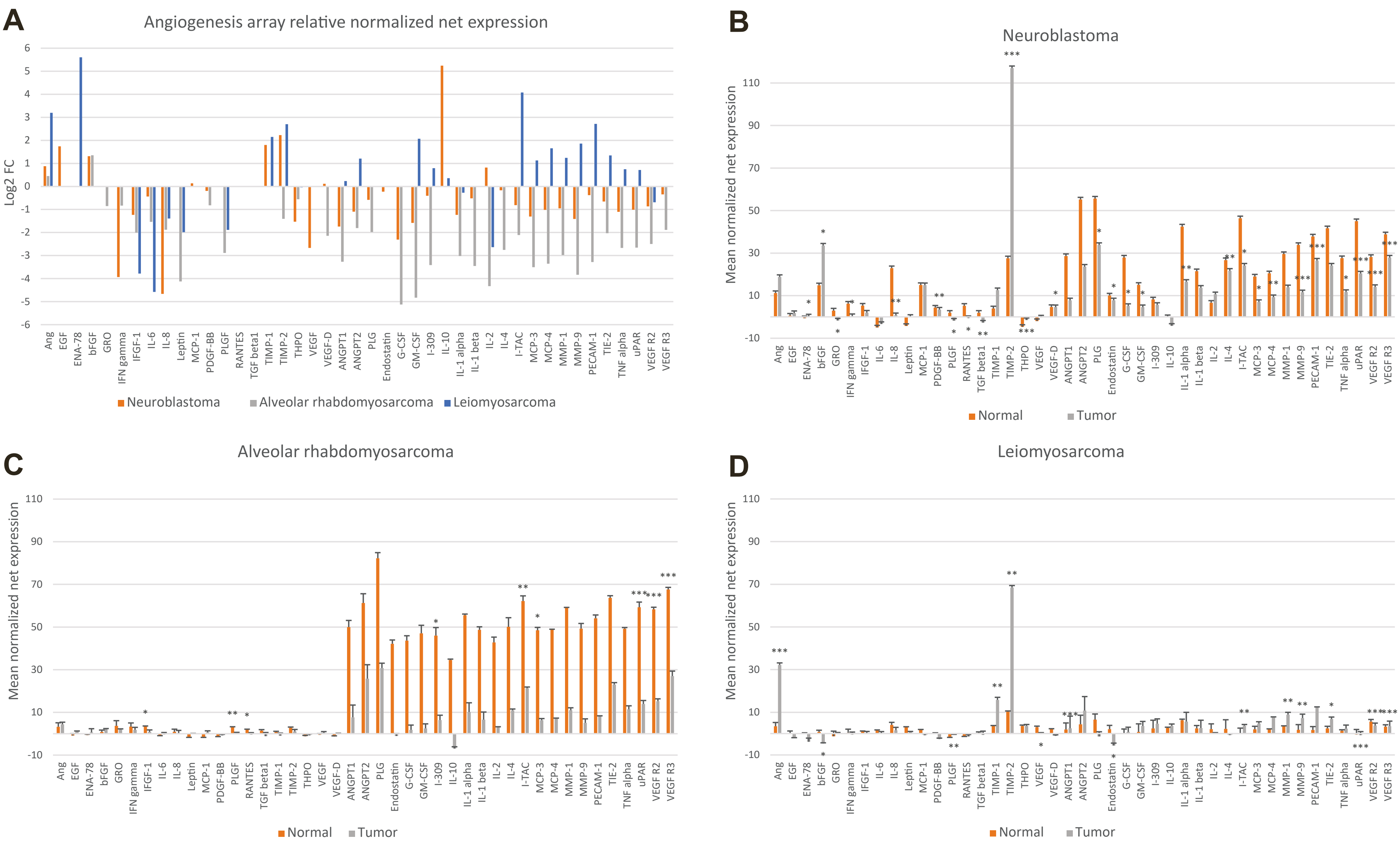

Fig S1. PRL3 expression is associated with elevation of pro angiogenesis factors. (A) Paired bar graph showing log2 fold-change of semi-quantitative intensity of Western blot signal of pro-angiogenic factors detected in pediatric tumor samples relative to matched adjacent normal tissue. Paired bar graphs of mean normalized semi-quantitative intensity of Western blot signal of pro-angiogenic factors in neuroblastoma (B), alveolar rhabdomyosarcoma (C) and leiomyosarcoma (D), with corresponding matched adjacent normal tissues.
