# Supplementary material for: Exploiting frequent and specific expression of PRL3 in pediatric solid tumors for first-in-child use of PRL3-zumab humanized antibody
Source: Mol Ther Oncolytics. 2023 Aug 18;30:153–66. doi: 10.1016/j.omto.2023.08.006 (PMC10477756; doi:10.1016/j.omto.2023.08.006)
Supplement: Document S2. Article plus supplemental information [file mmc2.pdf]

Molecular Therapy
Oncolytics
Original Article

Exploiting frequent and specific expression of PRL3 in pediatric solid tumors for first-in-child use of PRL3-zumab humanized antibody

Amos Hong Pheng Loh,[1,2,3] Min Thura,[4] Abhishek Gupta,[4] Sheng Hui Tan,[1] Kelvin Kam Yew Kuan,[4] Koon Hwee Ang,[4] Khurshid Merchant,[1,2,5] Kenneth Tou En Chang,[1,2,5] Hui Yi Yon,[5] Yong Chen,[2,3] Mathew Hern Wang Cheng,[6] Arjandas Mahadev,[2,6] Matthew Chau Hsien Ng,[2,7] Michaela Su-Fern Seng,[1,2,8] Prasad Iyer,[1,2,8] Pei Ling Chia,[4] Shui Yen Soh,[1,2,8] and Qi Zeng[4,9]

[1]VIVA-KKH Paediatric Brain and Solid Tumour Programme, Children's Blood and Cancer Centre, KK Women's and Children's Hospital Singapore 229899, Singapore; [2]Duke-NUS School of Medicine, Singapore 169857, Singapore; [3]Department of Paediatric Surgery, KK Women's and Children's Hospital, Singapore 229899, Singapore; [4]Institute of Molecular and Cell Biology (IMCB), Agency for Science, Technology, and Research (A*STAR), Singapore 138673, Singapore; [5]Department of Pathology and Laboratory Medicine, KK Women's and Children's Hospital, Singapore 229899, Singapore; [6]Department of Orthopaedic Surgery, KK Women's and Children's Hospital, Singapore 229899, Singapore; [7]Department of GI Oncology, National Cancer Centre Singapore, Singapore 229899, Singapore; [8]Department of Paediatric Subspecialties Haematology/Oncology Service, KK Women's and Children's Hospital, Singapore 229899, Singapore; [9]Department of Biochemistry, Yong Loo Lin School of Medicine, National University of Singapore, Singapore 119260, Singapore

Phosphatase of regenerating liver 3 (PRL3) is a specific tumor antigen overexpressed in a broad range of adult cancer types. However, its physiological expression in pediatric embryonal and mesenchymal tumors and its association with clinical outcomes in children is unknown. We sought to profile the expression of PRL3 in pediatric tumors in relation to survival outcomes, expression of angiogenesis markers, and G-protein-coupled receptor (GPCR)-mitogen-activated protein kinase (MAPK) signaling targets. PRL3-zumab, a first-in-class humanized antibody, was administered in a dose escalation schedule in a first-in-child clinical trial to study toxicity, pharmacokinetics, and clinical outcomes. Among 64 pediatric tumors, PRL3 was most frequently expressed in neuroblastoma (100%), rhabdomyosarcoma and non-rhabdomyosarcoma soft tissue sarcomas (71%), and renal sarcomas (60%) but absent in paired normal tissues. PRL3 was expressed in 75% of relapsed tumors and associated with shorter median event-free survival. Microarray profiling of PRL3-positive tumors showed elevation of angiogenin, TIMP1 and TIMP2, and GPCR-MAPK signaling proteins that commonly interacted with PRL3. The first use of PRL3-zumab in a pediatric patient saw no adverse events. A 28.6% reduction in maximum target lesion diameter was achieved when PRL3-zumab was administered concurrently with hypofractionated radiation. These findings support wider exploration of PRL3 expression in embryonal and mesenchymal tumors and further clinical application of PRL3-zumab in pediatric patients.

INTRODUCTION

Phosphatase of regenerating liver 3 (PRL3), encoded by *PTP4A3*, is a member of a class of dual-specificity protein tyrosine phosphatases consisting of three members: PRL-1, PRL-2, and PRL3.[1] PRL-3 is a C-terminal prenylated phosphatase induced in regenerating liver and was first found to be specifically overexpressed in colorectal cancer metastases.[2] Since then, PRL3 protein overexpression has been observed in a variety of cancers,[3–5] where it is generally associated with poorer prognosis and metastatic disease.[6,7] Our group and others previously described the role of PRL3 in oncogenic, metastasis, and angiogenic signaling.[8–11] PRL3 has been reported to be an excellent oncotarget, with expression in 80.6% of tumors across a broad range of cancer types but not in any adjacent normal tissue.[10,11] PRL3 is undetectable in the major normal human organs.[10] However, most studies have evaluated PRL3 expression in epithelial carcinomas more commonly found in adult patients, while the profile of PRL3 expression in pediatric cancers remains poorly understood. In Wilms tumor, the most common pediatric renal malignancy, PRL3 is associated with a 3.4-fold increase in risk of recurrence in favorable-histology disease.[12] However, in other childhood embryonal cancers and mesenchymal tumors, PRL3 expression and its relationship to clinical outcomes, such as relapse, have not been described.

Building on a unique concept of targeting intracellular oncoproteins with antibody therapies,[13] a humanized PRL3 antibody (PRL3-zumab;

Received 19 April 2023; accepted 15 August 2023;
https://doi.org/10.1016/j.omto.2023.08.006.

Correspondence: Amos Hong Pheng Loh, VIVA-KKH Paediatric Brain and Solid Tumour Programme, Children's Blood and Cancer Centre, KK Women's and Children's Hospital, Singapore 229899, Singapore.
E-mail: amos.loh.h.p@singhealth.com.sg
Correspondence: Qi Zeng, Institute of Molecular and Cell Biology (IMCB), Agency for Science, Technology, and Research (A*STAR), Singapore 138673, Singapore.
E-mail: mcbzengq@imcb.a-star.edu.sg

This is an open access article under the CC BY license (http://creativecommons.org/licenses/by/4.0/).

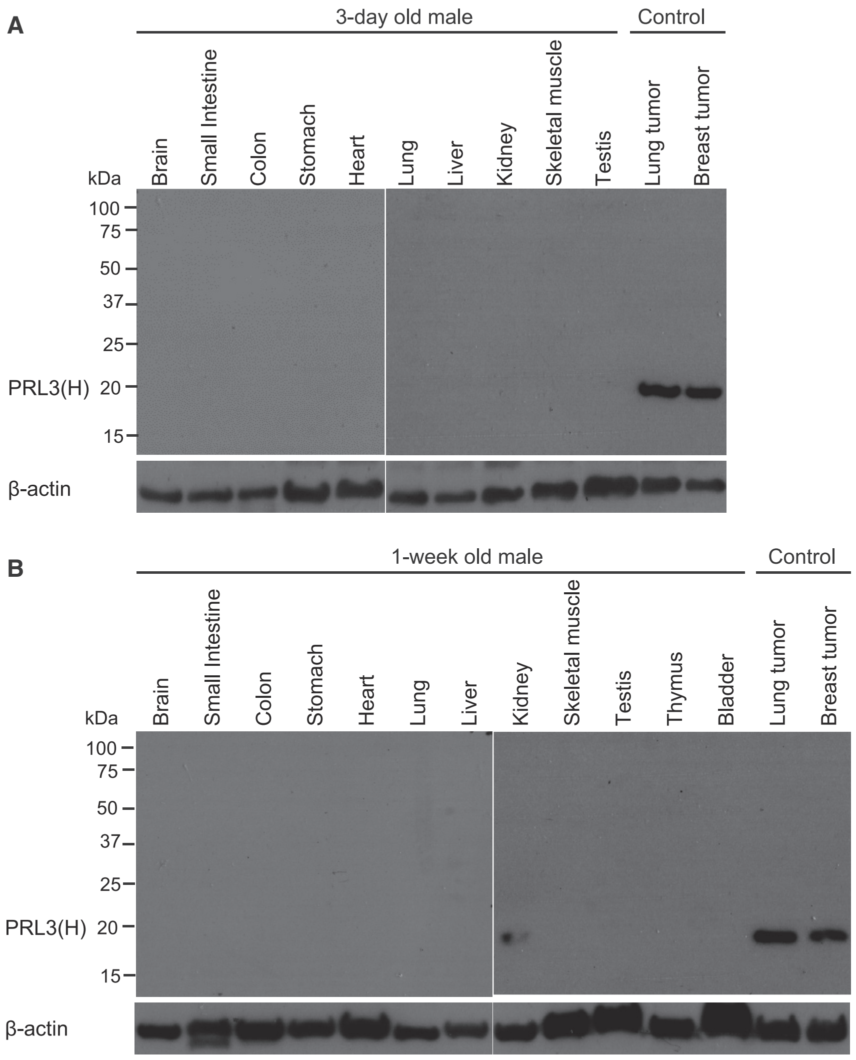

Figure 1. PRL3 is not expressed in a pediatric mouse normal tissue array

(A and B) Full western blotting of PRL3 expression in various organs of a (A) 3-day-old and (B) 1-week-old male FVB mice. The breast and lung tumor tissue from a 14-week-old female FVB-MMTV-PyMT mouse were used as positive controls. High PRL3 expression can be detected at 20 kDa in the positive control. Kidney tissue of 1-week-old mouse expressed weak PRL3. Blots were probed with an anti-PRL3 monoclonal antibody. B-actin was used as loading control.

immunoglobulin G1 [IgG1]) was developed and tested on a variety of human cancers using animal models.[14–16] To date, the agent has completed initial Phase I first-in-human testing in adults, demonstrating a good safety profile with no grade 3 or 4 drug-related severe adverse effects (SAEs) observed[17] and has progressed to phase II clinical trials (ClinicalTrials.gov: NCT04452955 and NCT04118114; Drugtrials.org.cn: CTR20211180).[5] These findings are consistent with *in vivo* safety assessments in murine and non-human primate models. This lack of toxicity in preclinical and clinical settings is due to the tumor-specific expression of PRL3 protein, which is largely absent in normal human adult tissues,[15] with PRL3 mRNA detected only in skeletal and cardiac muscle.[18,19] In preclinical studies, PRL3 has been shown to play a limited role in specification of neural crest progenitors[20] and regulates cellular migration in embryonic kidneys without affecting cell viability.[21,22] Therefore, we hypothesize that PRL3 may likewise be expressed in a highly specific manner in developmental cancers, facilitating safe application of PRL3-zumab in these diseases. However, PRL3 expression in the developing tissues of children and its toxicity profile in pediatric patients have not yet been described. In this study, we investigate the translational potential of PRL3 as a new therapeutic target in children to address the unmet clinical need for more targeted treatment of pediatric solid tumors.

RESULTS

PRL3 is overexpressed in a wide range of pediatric solid tumors and absent in normal tissues

While PRL3 oncoprotein is an established therapeutic tumor-specific oncotarget in mature adults, its baseline expression in developing tissues is not known. We have observed previously that PRL3 is undetectable in 13 major organs of adult FVB wild-type (WT) female mice but highly overexpressed in primary breast tumors and metastatic lung tumors of the spontaneous FVB/MMTV-PyMT tumor model.[14] To estimate PRL3 protein expression at human pediatric-equivalent developmental time points, we performed full immunoblots on protein lysates of various organs from 3-day- and 1-week-old male FVB/MMTV-PyMT mice, given the known interspecies correlation of organ development-related transcriptomes.[23] In contrast to control tumors from a 12-week-old female FVB/MMTV-PyMT mouse, PRL3 was largely absent in normal neonatal murine tissues, with only low expression seen in kidneys of the 1-week-old animal (Figure 1). This suggested that PRL3 was minimally expressed in developing organs in mice.

Next, we characterized PRL3 expression in pediatric solid tumors. Patients with newly diagnosed malignant solid tumors were recruited following informed consent, and excess tissue from initial tumor (primary), adjacent normal tissues, metastatic tumors, and relapse tumors was obtained and evaluated with full western blots (Table S1). In 64 specimens from 34 pediatric cancer patients, PRL3 was frequently expressed in pediatric malignant solid tumors and absent in matched normal tissues. Specifically, among embryonal tumors, PRL3 was present in neuroblastoma (7 of 7, 100%) and Wilms tumor (3 of 7, 43%) and absent in adjacent matched normal tissues

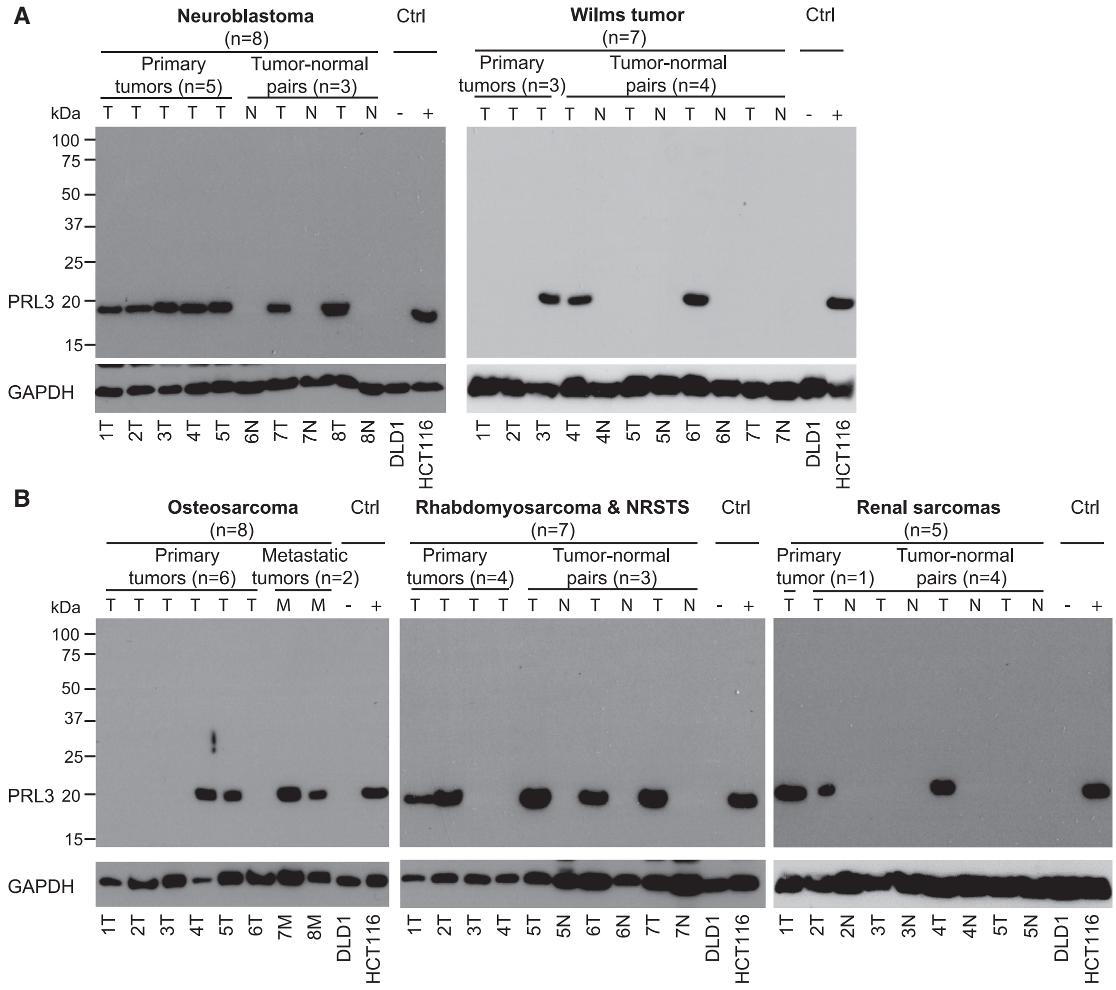

Figure 2. PRL3 is overexpressed in a wide range of pediatric solid tumors and absent in normal tissues

(A and B) Full western blot of PRL3 in pairs of primary tumor (T) and adjacent normal tissue (N) and metastatic tumors (M) from pediatric patients: (A) neuroblastoma and Wilms tumor and (B) osteosarcoma, rhabdomyosarcoma and non-rhabdomyosarcoma soft tissue sarcoma (NRSTS) and renal sarcoma. The DLD1 and HCT 116 human colon cancer cell lines were used as PRL3-negative and -positive controls (Ctrl). Blots were probed with an anti-PRL3 monoclonal antibody. GAPDH was used as a loading control.

(Figure 2A). Among pediatric sarcomas, PRL3 was present in osteosarcoma (4 of 8, 50%), rhabdomyosarcoma and non-rhabdomyosarcoma soft tissue sarcomas (NRSTSs) (5 of 7, 71%), and renal sarcomas of infancy (3 of 5, 60%) (Figure 2B). The data show that PRL3 protein was highly overexpressed in multiple pediatric solid tumors but not expressed in any of the adjacent normal tissues.

PRL3 is frequently expressed in pediatric solid tumors from patients with relapse and poor prognosis

Because PRL3 was frequently expressed in a wide range of pediatric solid tumors, we sought to determine whether its expression was also related to clinical characteristics in these patients. We first studied matched pairs of tumor samples from biopsies taken at initial diagnosis and relapse (Table S2). In tumor pairs from a patient with neuroblastoma and a patient with rhabdomyosarcoma, PRL3 was absent at initial diagnosis but expressed in the relapsed specimen. In two Wilms tumor pairs, PRL3 expression persisted from diagnosis to later relapse. Among three relapse osteosarcoma tumor pairs, PRL3 showed a mixed pattern of expression with detection at either time point in one pair and detection only at either initial diagnosis or relapse in the remaining two pairs. PRL3 expression could not be detected in the tumor pair from a patient with malignant mesothelioma. Overall, PRL3 was detected in 4 of 8 (50%) initial diagnostic biopsy samples and 6 of 8 (75%) relapsed tumor samples, suggesting that PRL3 overexpression is more frequently present in relapse samples than diagnostic samples (Figure 3A).

To understand the clinical characteristics associated with PRL3 expression, we compared PRL3 status among tumor types and in patients with metastatic versus localized disease. We also assessed the correlation between PRL3 status and event-free survival (EFS). Among 31 samples with complete clinical data available, PRL3 was

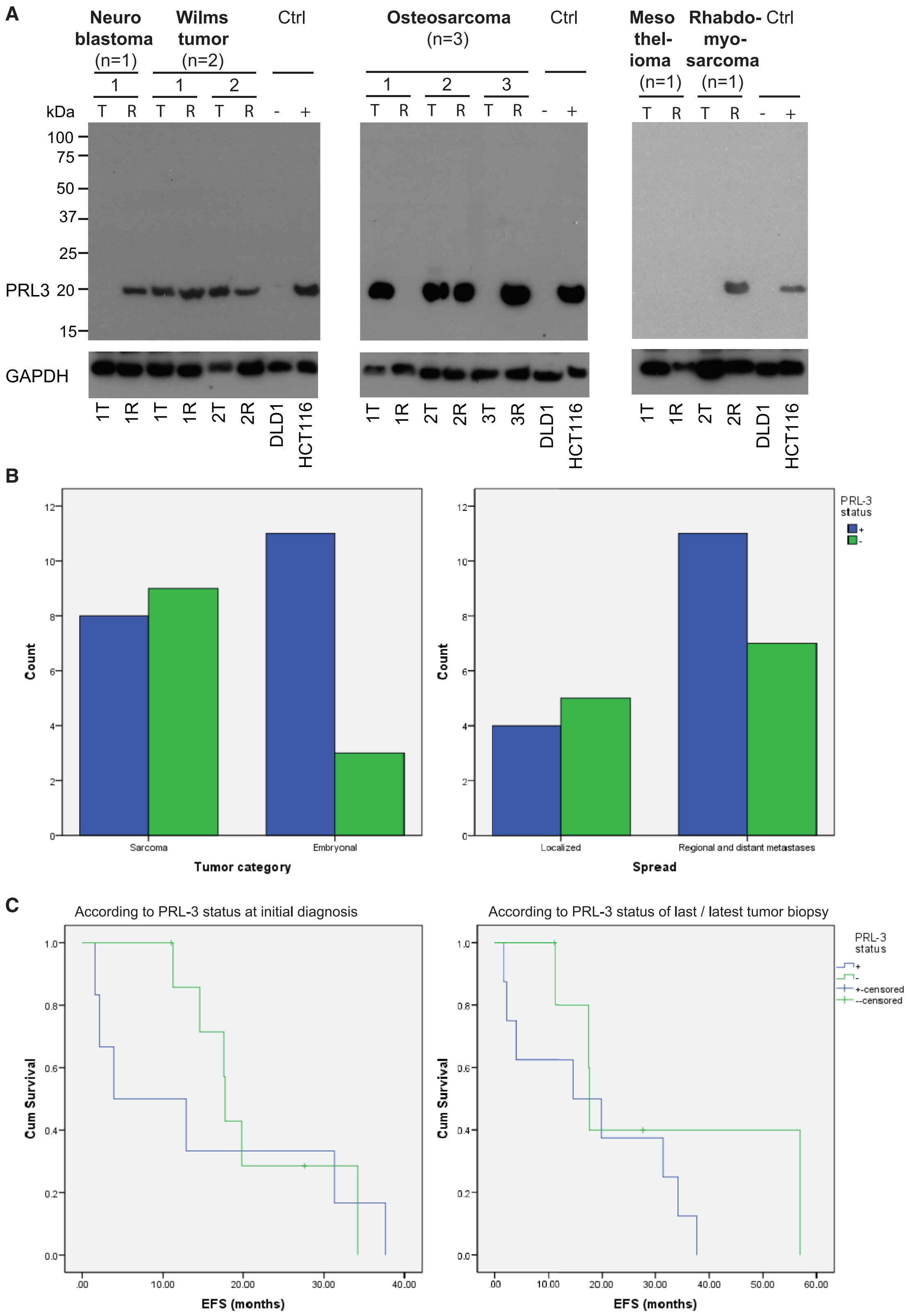

(legend on next page)

more frequently expressed in embryonal tumors (neuroblastoma and Wilms tumor) compared with sarcomas (osteosarcoma, rhabdomyosarcoma, NRSTSs, and renal sarcomas) as well as in patients with regional and distant metastases compared with localized disease (Figure 3B). In patients where survival data were available, EFS functions were estimated using the Kaplan-Meier method. The median estimated EFS duration was found to be shorter in patients with PRL3-positive tumors (Figure 3C). Notably, this shorter median estimated EFS was observed regardless of whether PRL3 status was determined by the initial diagnostic biopsy or the latest specimen in cases with serial surgical excisions. The median estimated EFS for PRL3-positive patients was 3.9 months compared with 17.7 months for PRL3-negative patients based on PRL3 status at initial diagnosis, while the median estimated EFS for PRL3-positive patients was 14.6 months compared with 17.7 months for PRL3-negative patients based on PRL3 status of the most recent tumor biopsy. This observation is in line with other studies that found higher PRL3 expression to be correlated with high-risk disease, metastasis, as well as poorer survival.[24–26] Thus, we anticipate that high PRL3 expression in tumor biopsies of pediatric tumors could be predictive of a poor prognosis.

PRL3 expression in pediatric tumor specimens is associated with elevation of pro-angiogenic factors and phosphorylation of G-protein-coupled receptor (GPCR)-mitogen-activated protein kinase (MAPK) pathway members

Because we observed PRL3 expression to be associated with poor prognostic features in a range of pediatric solid tumors, we next sought to understand which signaling pathways might be upregulated in PRL3-positive tumors, particularly angiogenic and GPCR-MAPK pathway targets that are known to be associated with PRL3 activation.[27,28] We interrogated five cases of PRL3-positive tumors using a human angiogenesis antibody array of 43 associated protein targets. Expression of angiogenin, TIMP1, and TIMP2 was specifically elevated in the PRL3-positive tumor specimens but not in PRL3-negative adjacent normal tissues (Figure 4A), with normalized net expression of these angiogenic factors increased in neuroblastoma, leiomyosarcoma, and rhabdomyosarcoma tumors (Figure S1). This observation was verified in corresponding full western blots using antibodies directed against these angiogenesis-related targets (Figure 4B). Consistent with these findings, PRL-3 is known to enhance tumor aggressiveness through regulation of matrix metalloproteinases, particularly via overexpression of angiogenin and invasion-associated enzymes of the MMP9-TIMP1/2 axis.[29,30]

Because PRL3 is also known to promote tyrosine and serine/threonine phosphorylation of diverse signaling proteins, particularly those of the GPCR-MAPK pathway, we sought to profile the phosphorylation status of these targets in relation to known PRL3 expression status. Tumor-normal pairs of representative cases of neuroblastoma (neuroblastoma 7T and 7N) and rhabdomyosarcoma (rhabdomyosarcoma 6T and 6N) were evaluated using a GPCR-MAPK microarray. Among 193 microarray targets, tumor expression of members of the cyclic AMP (cAMP)/protein kinase and oncogenic Ras/Raf protein families showed the greatest up-regulation in comparison with adjacent normal tissue (Figure 5A). Next, phosphorylation ratios of 84 proteins with non-phosphorylated and phosphorylated forms were determined and compared. In rhabdomyosarcoma, 30 proteins were phosphorylated and in neuroblastoma, 45 proteins were phosphorylated, with 16 proteins commonly phosphorylated in both (Figure 5B). To understand the association of these phosphoproteins with PRL3, a STRING protein-protein association network was constructed, comprising these commonly phosphorylated proteins along with PRL3. PRL3 interacted with 14 of these proteins via Src (Figure 5C), a known association in multiple other cancers.[31–35] Our findings of frequent phosphorylation of GPCR-MAPK protein targets in pediatric tumors reflects data from adult cancers suggesting that PRL-3 may promote phosphorylation of multiple oncogenic proteins by functioning as a possible activator kinase.[27]

First-in-child compassionate use trial of PRL3-zumab in combination with radiotherapy for a patient with recurrent metastatic rhabdomyosarcoma

PRL3-zumab is a first-in-class humanized antibody drug that specifically targets PRL3-overexpressing tumors without damaging surrounding healthy tissues, targeting PRL3 externalized on the tumor cell surface.[14,15] Given its established safety profile from the initial phase I trial[17] and ongoing phase II trials in adult patients with advanced solid tumors, PRL3-zumab was administered to a pediatric patient with multiply relapsed metastatic *PAX7-FKHR* fusion-positive rhabdomyosarcoma on a compassionate basis after diagnosis of a fifth relapse and disease progression. The prior treatment history from initial diagnosis to fourth relapse is described in Table S3. First, to establish baseline and current expression of PRL3, samples of the patient's primary tumor and two subsequent relapse tumors were assessed for expression of PRL3 by western blotting. PRL3 was not detected in the primary tumor, but high expression was detected in the first and second relapsed tumors (Figure 6A). Given these findings, and serial recurrence despite multiple lines of treatment, PRL3-zumab was added to the salvage treatment plan for the fifth relapse (Table S4). While awaiting commencement of PRL3-zumab treatment, the patient received two doses of bridging salvage chemotherapy with intravenous (i.v.) vinorelbine given 7 days apart.

Figure 3. PRL3 is frequently expressed in pediatric solid tumors with relapse and poor prognostic features
(A) Full western blot of PRL3 in initially diagnosed primary tumor (T) and relapsed tumor (R) tissue pairs from malignant pediatric tumors: neuroblastoma, Wilms tumor, osteosarcoma, mesothelioma, and rhabdomyosarcoma. DLD1 and HCT 116 cells were used as negative and positive controls (Ctrls), respectively. GAPDH was used as a loading Ctrl. (B) Comparison of PRL3 expression based on tumor type (sarcomas [osteosarcoma, rhabdomyosarcoma, NRSTS, and renal sarcomas] and embryonal tumors [neuroblastoma and Wilms tumor] (left panel, n = 34, p = 0.073, chi-square test) and based on the spread of tumor (right panel, n = 27, p = 0.411, chi-square test). (C) Event-free survival (EFS) was compared using the Kaplan-Meier method according to PRL3 status at initial diagnosis (left panel) and according to PRL3 status of last/latest tumor biopsy (right panel). Shorter median estimated EFS was observed in patients with PRL3-positive tumors.

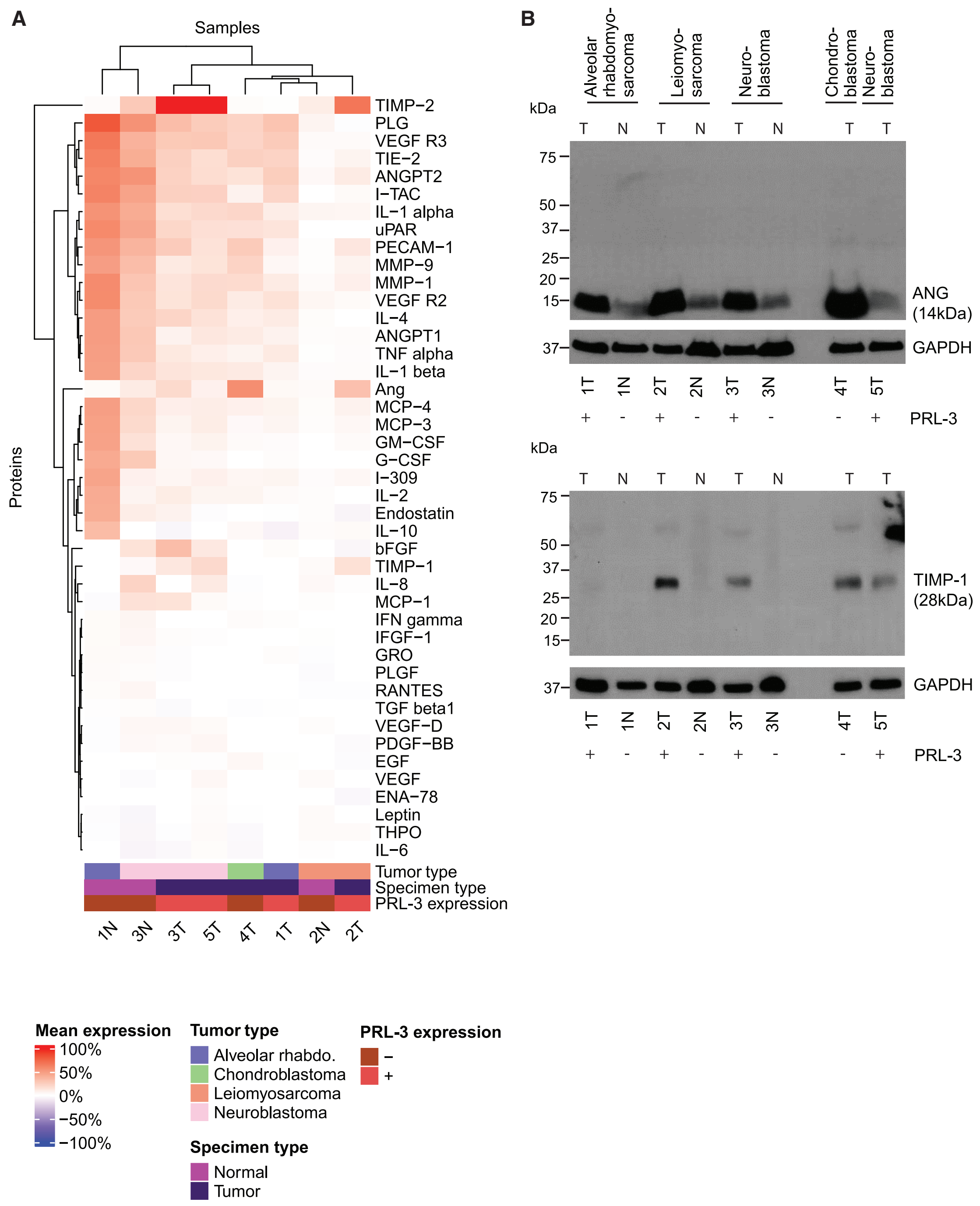

Figure 4. PRL3 expression is associated with elevation of the pro-angiogenic factors angiogenin, TIMP-1, and TIMP-2 in multiple pediatric solid tumors
(A) Heatmap displaying unsupervised clustering of mean semiquantitative expression of pro-angiogenic factors detected on an antibody array applied to pediatric tumor samples and corresponding matched adjacent normal tissues. Expression of angiogenin, TIMP-1, and TIMP-2 was uniquely elevated in PRL3-positive tumor specimens but not in PRL3-negative normal adjacent tissues. (B) Verification of the observation in (A) by corresponding full western blots using directed antibodies against these pro-angiogenic targets to show angiogenin and TIMP-1 protein levels.

Subsequently, over 1 month, two sessions of hypofractionated palliative radiotherapy (RT; 2 × 4 Gy) to the left chest tumors were given over two consecutive days each, prior to and concurrent with treatment with PRL3-zumab. Beginning at a dose range below the established recommended phase 2 dose for adults of 6 mg/kg every 2 weeks, PRL3-zumab was administered initially in incremental doses from 4

A

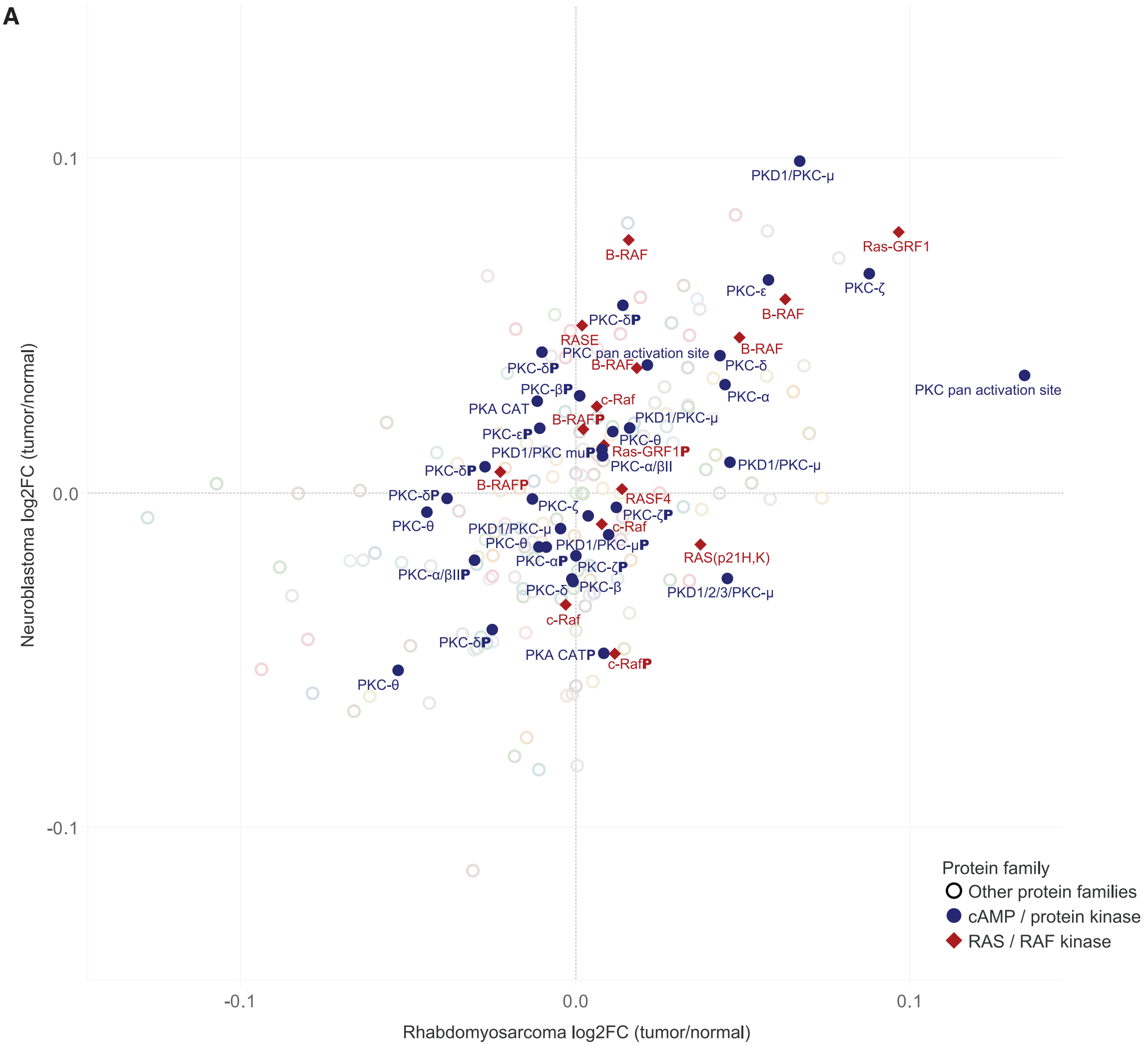

B

Phosphorylated proteins *(phosphorylation ratio >1)*	Rhabdo-myo-sarcoma	Neuro-blastoma
Arrestin-1 (Phospho-Ser412)	1.01	1.07
B-Raf (Phospho-Ser446)	1.03	1.07
CDC25B (Phospho-Ser323)	1.01	1.01
CDC25B (Phospho-Ser353)	1.05	1.03
DYN1 (Phospho-Ser774)	1.09	1.08
GRK2 (Phospho-Ser29)	1.01	1.03
MAPKAPK2 (Phospho-Thr334)	1.02	1.05
MEK1 (Phospho-Ser298)	1.01	1.01
MKP-1/2 (Phospho-Ser296)	1.08	1.07
PKA CAT (Phospho-Thr197)	1.08	1.15
PKC pan activation site (Phospho)	1.04	1.01
PKD1/PKC mu (Phospho-Tyr463)	1.04	1.02
PKD1/PKC mu (Phospho-Ser910)	1.02	1.07
RASGRF1 (Phospho-Tyr504)	1.08	1.27
Src (Phospho-Ser75)	1.05	1.11
Tyrosine Hydroxylase (Phospho-Ser8)	1.04	1.17

C

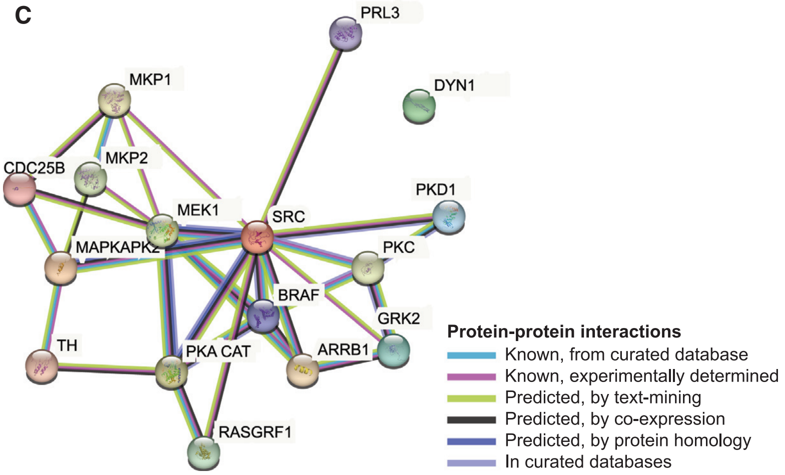

(legend on next page)

to 5.4 mg/kg/day, with the intention to increase gradually to the full adult dose (Table S4). As a precautionary measure for this first-in-child use of PRL3-zumab, the first dose was administered in two divided doses, while a shorter dosing interval of 8–10 days was used in view of the patient's advanced disease (Figure 6B; Tables S3–S5).

Safety and toxicity

Throughout the trial, the patient did not experience any inflammatory responses, adverse effects, or SAEs and tolerated the infusion very well even as PRL3-zumab was progressively increased to the full dose. The laboratory parameters remained stable after treatment with PRL3-zumab. Significant reduction of hemoglobin after chemotherapy and RT were restored to initial levels within a week. White blood cell (WBC) and neutrophil counts remained within the normal range. Absolute lymphocyte counts were low, which was likely due to prior chemotherapy and RT. Liver enzymes were within the normal range, apart from consistently elevated alkaline phosphatase (Table S6).

Quality of life

The patient weighed 14.2 kg at the start of the compassionate trial. The Lansky scale was 70, and Eastern Cooperative Oncology Group (ECOG) performance status was 1. The patient displayed mild respiratory symptoms and required oxygen at night, but other organ functions were normal. A week after receiving the first dose, the patient remained clinically well, weighed 14.3 kg, was weaned off oxygen, no longer required morphine because pain had subsided, and could be nursed at home. Five days after the second dose of PRL3-zumab, the patient developed an intermittent left frontal headache but with no abnormalities on physical examination. The symptoms did not interfere with daily activities, and the patient was able to attend school for 3 days during that time and had also gained 0.4 kg. The patient was given paracetamol for relief and still did not require opiates or supplemental oxygen.

Efficacy

Following bridging chemotherapy and two doses of PRL3-zumab with hypofractionated RT, serum lactate dehydrogenase (LDH) levels decreased to a nadir of 212 U/L from the prior baseline of 315 U/L prior to the fifth relapse, suggesting clinical response to the multimodal therapy. Subsequently, LDH levels increased to a peak of 614 U/L after cessation of the trial, corresponding to the observed development of new progressive disease (Figure 6B, top panel). Serial chest computed tomography (CT) and whole-body PET/CT scans were taken for periodic efficacy assessment. Maximum target lesion (TL) diameter measured 4.9 cm at baseline prior to the fifth relapse and was reduced to 3.5 cm after initiation of the multimodal regimen incorporating PRL3-zumab—a reduction of 28.6% (Figure 6B, top panel). While the left chest TL showed improvement (Figure 6B, bottom panel), lesions in the right chest (non-TLs) showed progression, and new metastatic disease in the left eye orbit and liver were detected. According to Response Evaluation Criteria in Solid Tumours (RECIST) 1.1 criteria, this response was categorized as stable disease (<30% reduction in maximum TL diameter). Thus, while the combination of RT followed by PRL3-zumab led to tumor reduction in the left chest, because of the onset of new distant disease, the salvage treatment was stopped in lieu of palliative care.

Pharmacokinetics

Pharmacokinetics (PK) samples were collected pre and post C1D1, C1D8, and C2D1 doses. Serum concentrations of PRL3-zumab at each time point were detected using ELISA, and non-compartmental model analysis of PK parameters for single-dose administration was performed. The patient C_{max} was 71.23 μg/mL, area under curve (AUC) was 27,130.25 μg/mL h, and $t_{1/2}$ was 10.9 days (Figure 6C). The serum concentration and PK parameters of this pediatric patient were comparable with those of an adult patient administered a 6 mg/kg dose in the phase I clinical trial (Figure 6D).

In summary, in this first pediatric patient treated with PRL3-zumab on a compassionate dose escalation protocol, treatment was well tolerated with short-term improvement in quality of life and reduction of TL size. Unfortunately, because of development of distant metastatic disease, treatment had to be discontinued in pursuit of palliative care options.

DISCUSSION

In this study, we found PRL3 to be expressed in a wide variety of pediatric solid tumors at diagnosis and relapse but not in normal patient tissues and developing organs of neonatal mice. Pediatric patients with tumor expression of PRL3 more frequently displayed negative prognostic clinical characteristics; PRL3-expressing pediatric tumors also showed overexpression of angiogenic factors and cancer-associated kinases implicated in other PRL3-positive cancers. Together, the preclinical evidence indicated PRL3 to be a prevalent and specific cancer target in childhood tumors, which supported the compassionate first-in-child use of PRL3-zumab in a pediatric patient with multiply recurrent rhabdomyosarcoma. The dose escalation trial had no drug-related SAEs and yielded short-term responses in serum

Figure 5. Microarray of expression of GPCR-associated proteins in pediatric solid tumors demonstrates phosphorylation of protein kinase and Ras/Raf proteins, which are associated with PRL3

(A) Scatterplot of mean normalized relative expression of GPCR microarray targets in tumor-normal pairs of representative cases of neuroblastoma (neuroblastoma 7T and 7N) and rhabdomyosarcoma (rhabdomyosarcoma 6T and 6N); members of the two protein families showing greatest up-regulation in tumor versus normal tissue are highlighted (protein kinase/cAMP, blue; Ras/Raf, red). (B) GPCR-associated proteins commonly phosphorylated in tumor samples of the representative rhabdomyosarcoma and neuroblastoma cases (phosphorylation is defined as phosphorylation ratio > 1). (C) STRING protein-protein association network of commonly phosphorylated proteins of representative pediatric solid tumors (from B) alongside PRL3, with known and predicted protein-protein interactions indicated by colored lines. Notably, PRL3 shares direct associations with members of the protein kinase and Ras/Raf families, which are commonly phosphorylated in pediatric rhabdomyosarcoma and neuroblastoma.

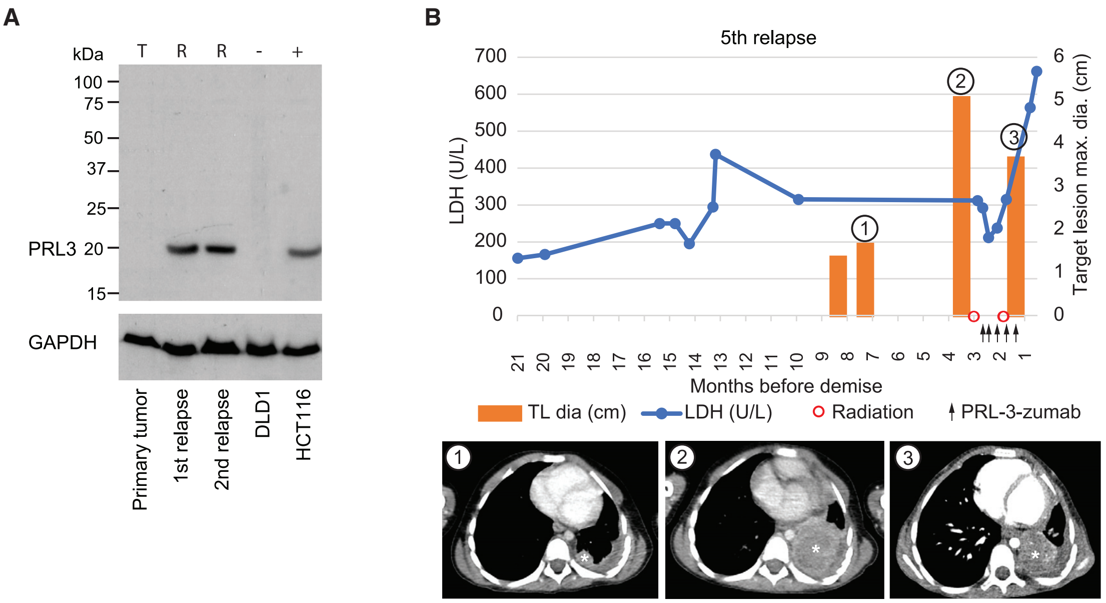

C

PK parameters		
**Parameter**	**Unit**	**Value**
t1/2	days	10.99
CL	(mg)/(μg/ml)/	0.0001
Cmax	μg/ml	71.23
Css	μg/ml	27130.25
AUC 0-t	μg/ml*h	10731.68
AUC 0-∞	μg/ml*h	27130.25
Vss	mg/(μg/ml)	0.06

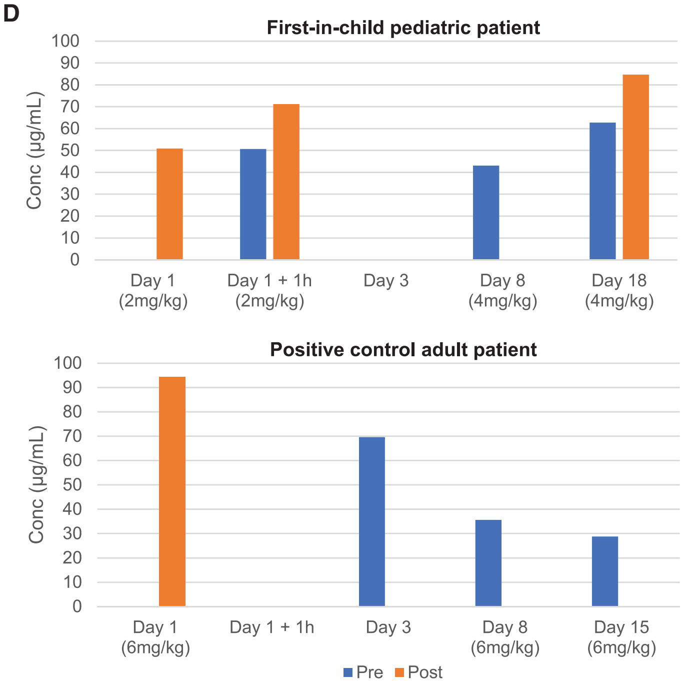

Figure 6. First-in-child compassionate clinical trial of PRL3-zumab in combination with chemotherapy and RT shows a reduction of the TL and a PK profile similar to adult data

(A) Full western blot of PRL3 expression of biopsies of primary tumor at initial diagnosis (T) and first and second relapses (R). The DLD1 and HCT116 cell lines were used as negative and positive Ctrls of PRL3 protein expression, respectively; GAPDH is used as a loading Ctrl. (B) Multiple parameter plot showing serum LDH level and target lesion (TL) size over 21 months leading up to terminal demise. A blue line indicates the LDH level, orange bars indicate TL size, red circles indicate hypofractionated palliative RT sessions, and black arrows indicate the times when PRL3-zumab therapy was given. Encircled numerals indicate the representative contrast-enhanced axial CT images in the corresponding lower panel: (1) at conclusion of treatment of the fourth relapse, (2) at diagnosis of the fifth relapse, and (3) after conclusion of RT and PRL3-zumab therapy. Asterisks in CT images indicate TL nominated for iRECIST primary endpoint determination. (C) PK parameters calculated based on non-compartmental model single-dose therapy. (D) Serum concentration of PRL3-zumab in the first-in-child PRL3-zumab trial patient (top panel) compared with the serum concentration of PRL3-zumab in an adult patient (bottom panel).

markers and TL size. Though the treatment was initiated in the context of very advanced disease, PRL3-zumab demonstrated drug efficacy after the first dose in the pediatric patient with metastatic and recurrent rhabdomyosarcoma. The patient showed improved quality of life; he did not require oxygen supplementation or morphine, experienced weight gain, and was able to resume some daily activities, such as attending school for 3 days. It is likely that the drug efficacy was due to the combination of PRL3-zumab with RT.

Overall, PRL3-zumab stabilized disease in the pediatric patient while providing better quality of life, and with minimal side effects. These findings indicate the clinical potential of PRL3 as a therapeutic target and prognostic biomarker in pediatric solid tumors and the safety and efficacy of its corresponding antibody drug PRL3-zumab in this patient population.

Pediatric solid tumors comprise just over half of all pediatric cancers but account for a disproportionately greater percentage of childhood cancer-related deaths.[36–39] Most notably, survival rates for pediatric brain and bone cancers and soft tissue sarcomas have plateaued in the last 2–3 decades,[36,40] with the least advancements seen in metastatic bone and soft tissue sarcomas.[41] This may represent the limit of therapeutic gains from treatment intensification strategies,[42] considering also the acute burden of symptom distress and, in survivors of childhood cancer, late effects that substantially impair quality of life.[43,44] These trends are concerning given the proliferation of pediatric precision oncology trials in recent decades, which, despite having facilitated increased identification of targeted therapeutic options, have also encountered low rates of treatment assignment because of the low frequency of actionable molecular alterations in childhood tumors.[45,46] To date, kinase inhibitors targeting ALK-, TRK-, and ROS-rearranged tumors[46–49] and ALK-, RET-, and BRAF-mutated tumors,[50–52] have shown the most success in pediatric brain and solid tumors with objective response rates of greater than 70%, but numbers of eligible patients are few. For example, NTRK fusions are found in only 3% of pediatric tumors[53] and ALK in 8%–12% of neuroblastoma.[54,55] Even with novel trial strategies constraining precision therapeutics only to relapse/refractory disease, incremental yield from these interventions remains limited.[56–58] Hence, there remains an urgent need for more effective but safe treatments for pediatric tumors, particularly sarcomas, relapse, and metastatic disease.

A major barrier limiting the development of cancer therapeutics is the intracellular location of most oncogenic driver proteins, which has traditionally precluded effective targeting of surface targets.[59] In pediatric tumors, their low mutational burden further limits the number of altered surface proteins for neoantigen-based therapeutic strategies.[60] Adopting an alternative strategy of targeting intracellular proteins with a humanized antibody could address this limitation. PRL3-zumab has demonstrated preclinical efficacy against a wide variety of cancers expressing PRL3.[14,15,59,61,62] Given the broad range of pediatric solid tumor types demonstrated in this study to also express this oncoprotein, this makes PRL3 an attractive novel therapeutic target for pediatric tumors. Notably, proof of this new strategy of targeting intracellular oncoproteins has also been demonstrated preclinically with chimeric antigen receptor (CAR) T cells) targeting major histocompatibility complex (MHC) peptides from intracellular oncoproteins such as PHOX2B and WT1.[60,63–65] The safety data emerging from ongoing clinical trials with PRL3-zumab in adult patients has shown no drug-related SAEs (ClinicalTrials.gov: NCT03191682, NCT04452955, and NCT04118114; Drugtrials.org.cn: CTR20211 180).[5] Our clinical experience from first-in-child use of PRL3-zumab also suggests that the agent should have a similarly favorable safety profile in children and potential to positively impact quality of life and tumor response. Therefore, further clinical studies in younger patients could be pursued to evaluate the utility of PRL3-zumab in pediatric tumor patients.

PRL3 expression has been associated with inferior prognosis in multiple epithelial carcinomas[4,5,24–26,66] and characterized as an activator of multiple kinase signaling pathways, especially phosphatidylinositol 3-kinase (PI3K)/AKT, MAPK/ERK, and EGFR.[6,7,27,67,68] This first study in pediatric tumors now extends the scope of diseases where PRL3 expression is negatively associated with clinical prognosis. We also explored possible oncogenic mechanisms that might be responsible for the adverse behavior in childhood tumors and found that Src was commonly phosphorylated in neuroblastoma and rhabdomyosarcoma tumors. While this has also been described in other adult tumors expressing PRL3,[31–35] interestingly it was in human embryonic kidney cells where PRL3 was first observed to mediate Src activation via Csk down-regulation.[31] Notably, the degree of Csk activity is positively correlated with ectodermal differentiation in embryonic stem cells and neurogenic differentiation in neuroblastoma,[69–71] though its role in regulating cellular differentiation in other developmental cancers is not known. These findings point to a need for further study of the mechanistic role of PRL3 signaling in embryonal tumors, particularly in relation to crosstalk with G-protein-coupled receptors and Src-family kinases. Another area that warrants future study is expression of PRL3 in mesenchymal tumors, particularly rhabdomyosarcoma and osteosarcoma, where there is currently a dearth of clinical and preclinical evidence. Also, given the rarity of pediatric cancers and the variety of different histological subtypes, larger profiling studies are required to validate our findings.

We found that PRL-3 was a cancer-specific marker frequently expressed in a wide variety of pediatric solid tumors, particularly neuroblastoma, osteosarcoma, and rhabdomyosarcoma. In the first clinical experience with PRL3-zumab in a pediatric patient, PRL3-zumab again demonstrated a good safety profile and yielded short-term disease stabilization and improvement of quality of life when used in combination with hypofractionated RT. Further characterization of PRL3 expression in embryonal and mesenchymal tumors is needed, along with wider clinical experience with use of PRL3-zumab in children, possibly in the context of basket trials for pediatric cancers.

MATERIALS AND METHODS

Patients and specimens

From 2015–2022, all pediatric patients with malignant solid tumors who were undergoing surgical biopsies or resections were prospectively recruited at KK Women's and Children's Hospital with institutional review board approval (protocols 2012/450 and 2014/2079, "Modeling, Analysis and Translational Therapeutics for Tumors of Childhood [MAT3CH]"). Written consent was obtained from parents and assent from children. Where available, aliquots of excess tumor tissue from these routine surgical procedures were snap frozen for molecular analysis. Disease characteristics and survival outcome data were directly recorded or obtained from the Singapore Childhood Cancer Registry. Additional archival frozen tissues were obtained from the SingHealth Tissue Repository.

Animals

Animal study was performed in accordance with approved guidelines with Agency for Science, Technology, and Research (A*STAR) institutional animal care and use committee (IACUC) approval (IACUC 161130). Tissue arrays of 3-day- and 1-week-old FVB mice and 14-week-old Mouse Mammary Tumor Virus-Polyoma virus Middle T antigen (MMTV-PyMT) mice were collected after euthanasia.

Protein extraction and western blotting

Protein extraction

Protein extraction and immunoblotting were performed as described previously. 100 mg of tissue was suspended in 50 μL of radioimmunoprecipitation assay (RIPA) lysis buffer (Sigma) supplemented with a protease and phosphatase inhibitor cocktail (Roche) for 15 min at 4°C and homogenized by tissue homogenizer (Polytron). The supernatant of tissue lysates was collected after centrifugation at 13,000 × *g* for 40 min at 4°C. For cultured cells, 5×10^6 cells were lysed in lysis buffer and clarified as described above. Protein concentrations of tissue and cell lysates were estimated using a bicinchoninic assay kit (Pierce). After addition of 2× Laemmli buffer containing dithiothreitol (DTT) (50 mM final concentration), samples were boiled and used immediately for western blotting or stored at −80°C until use.

Western blotting

Tissue and cell lysates were resolved on 14% SDS-polyacrylamide gels and transferred to nitrocellulose membranes before blocking and probing with the indicated primary antibodies at 1:2,000 dilution (PRL3) or 1:100,000 dilution (GAPDH) overnight at 4°C. After thorough washing with TBS-T buffer (20 mM Tris [pH 7.6], 140 mM NaCl, 0.2% Tween 20), the membranes were incubated with goat anti-mouse H+L IgG horseradish peroxidase (HRP)-conjugated secondary antibodies at a 1:5,000 dilution for 1 h, washed with TBS-T, and visualized using a chemiluminescent substrate (Millipore).

Angiogenesis array

Protein samples from tumor and matched normal tissues were used in the angiogenesis array. The angiogenesis array was performed according to the manufacturer's instructions (ab193655). In brief, each membrane was blocked with 1× blocking buffer at room temperature for 30 min. After aspiration, 50 μg of protein samples (diluted in 1 mL of 1× blocking buffer) was added onto the membranes and incubated overnight at 4°C. The following day, the membranes were washed three times with 1× wash buffer I and incubated for 5 min each at room temperature. The membranes were further washed three times with 1× wash buffer II for 5 min each. Subsequently, the membranes were incubated with 1× biotinylated antibody cocktail for 2 h at room temperature. Then the membranes were washed three times with 1× washing buffer I and three times with 1× washing buffer II. 1× HRP-conjugated streptavidin was added to the membranes, which were then incubated for 2 h at room temperature. Membranes were washed three times with 1× wash buffer I and three times with 1× washing buffer II. Finally, the membranes were visualized using the chemiluminescence substrate provided in the kit. The density of each spot was quantified using ImageJ (1.53C). The background signal was subtracted from the raw densitometry data, and the resultant data were normalized to the positive control. The expression levels of cytokines were compared between tumor and matched normal tissue samples.

GPCR microarray

Protein lysates obtained from selected frozen samples of tumor and matched normal tissues were applied to a GPCR-MAPK Pathway Phosphorylation Antibody Array (Full Moon BioSystems, USA) containing antibodies against 193 targets in 6 replicates printed on standard-size coated glass slides. Briefly, the arrays were first blocked with blocking solution (Full Moon BioSystems) for 30 min at room temperature and then incubated with the biotin-labeled cell lysates at 4°C overnight. After washing 3 times, the conjugated labeled proteins were detected using Cy3-conjugated streptavidin (S32355 streptavidin, Alexa Fluor 555 conjugate, Invitrogen). Hybridized array slides were scanned with a SureScan microarray scanner (G2600D, Agilent Technologies, Santa Clara, CA, USA) at 10 μm per pixel, and the single-channel image data were extracted and converted to Elist object files using Agilent Feature Extraction software. Read data were processed using limma on R (v.4.1.2) as follows. Normalization was performed using adaptive background correction, normalized between arrays, and averaged across duplicate spots. For quality control, raw intensities and read spot types were checked, and between-array MA plots (log intensity ratios vs. log intensity averages) and comparative density plots across arrays were created and inspected; also, sigma vs. A mean plots (residual variances vs. average log expression) were used to check the mean-variance relationship of the expression data. Fold change and standard error were estimated by fitting a linear model for each protein target, following averaging across duplicate spots, and then used to compute the phosphorylation ratio of phosphorylated and unphosphorylated protein pairs, as follows: phosphorylation ratio = $(\text{phospho}_{\text{tumor}}/\text{unphospho}_{\text{tumor}})/(\text{phospho}_{\text{normal}}/\text{unphospho}_{\text{normal}})$. Protein-protein interactions of commonly phosphorylated proteins and PRL3 in pediatric solid tumors were evaluated using STRING v.11.5.[72]

First-in-child compassionate use trial

The first-in-child compassionate use trial of PRL3-zumab was conducted at KK Women's and Children's Hospital with written parental

consent and child assent. In this custom protocol, 2 sessions each of hypofractionated palliative RT were given over 2 consecutive days before and during the PRL3-zumab treatment. The C1D1 dose of PRL3-zumab was commenced 10 days after the initial 2 RT sessions. The drug was administered intravenously in 2 divided doses that were 6 h apart, each at 2 mg/kg, with a total of 56 mg; a full standard dose of PRL3-zumab is 6 mg/kg. The C1D8 dose of PRL3-zumab was administered as a single infusion at 4 mg/kg with a total of 57 mg. C2D1 was administered 10 days later at a dose of 4.8 mg/kg with a total of 70 mg, and C2D11 was administered at a dose of 5.4 mg/kg with a total of 80 mg. The primary endpoint was progression-free survival (PFS) according to RECIST 1.1 and iRECIST criteria.[73] Secondary endpoints were overall survival (OS) and duration of response according to RECIST and iRECIST criteria. Safety was assessed by Common Terminology Criteria for Adverse Events (CTCAE) version 5 criteria with adverse event (AE), serious adverse event (SAE), vital signs, ECOG performance status (PS), physical examination, and clinical laboratory parameters. quality of life (QoL) was assessed based on general patient wellbeing and body weight.

PK analysis of PRL3-zumab in the pediatric clinical trial patient

PK samples were collected pre and post dose of C1D1 (a.m. and p.m.), C1D8, and C2D1. ELISA was performed to detect the concentration of PRL3-zumab in serum. Briefly, 96-well plates coated overnight with glutathione S-transferase (GST)-PRL3 (1 ng) were blocked with 3% bovine serum albumin in PBS-0.05% Tween 20 prior to incubation with a serum sample (1:20,000) for 1 h at 37°C. After extensive washing, an HRP-conjugated anti-human antibody (Pierce) was added for 1 h at 37°C. Colorimetric development was performed using a Turbo-TMB substrate (Pierce) and stopped by acidification with 2 M H_2SO_4. Absorbance was measured at 450 nm using a plate reader (Tecan). Non-compartmental model analysis of PK parameters for single-dose administration was performed using PK Solver software. PK parameters for PRL3-zumab including C_{max}, AUCinf, and $t^1/_2$ were analyzed.

DATA AND CODE AVAILABILITY

The datasets supporting the conclusions of this article are included within the article and its additional files.

SUPPLEMENTAL INFORMATION

Supplemental information can be found online at https://doi.org/10.1016/j.omto.2023.08.006.

ACKNOWLEDGMENTS

The authors thank Dr. Min Hwee Yong, Chik Hong Kuick, Eileen Hui Qi Ng, Chun Lin Lye, and Germaine Liew, KK Women's and Children's Hospital, for research administrative support. This work was supported by the VIVA Foundation for Children with Cancer and the KKH Health Fund (grant 02/FY2015/EX/06-A42 [VIVA-KKH Paediatric Brain and Solid Tumour Programme]) to A.H.P.L. and the IMCB A*STAR core fund to Q.Z.

AUTHOR CONTRIBUTIONS

A.H.P.L. and Q.Z. designed the experiment and manuscript. A.H.P.L., S.H.T., K.M., K.T.E.C., Y.C., M.H.W.C., A.M., M.S.-F.S., P.I., and S.Y.S. provided clinical material. S.Y.S., M.C.H.N., and Q.Z. designed the clinical trial. S.Y.S. conducted the clinical trial. A.H.P.L., A.G., K.K.Y.K., K.H.A., H.Y.Y., and M.T. performed and analyzed ECL, ELISA, and microarray data. S.Y.S. and A.H.P.L. analyzed clinical trial data. M.T. designed and analyzed PK data. A.H.P.L., P.L.C., K.H.A., M.T., and Q.Z. prepared, reviewed, proofread, and finalized the manuscript. All authors read and approved the final manuscript.

DECLARATION OF INTERESTS

Q.Z. is the founder of Intra-Immu SG Pte Ltd., an A*STAR spin-off company granted licensing rights to the PRL3-zumab IP portfolio. Intra-Immu SG Pte Ltd. provided PRL3-zumab used in this study.

REFERENCES

1. Gao, P.P., Qi, X.W., Sun, N., Sun, Y.Y., Zhang, Y., Tan, X.N., Ding, J., Han, F., and Zhang, Y. (2021). The emerging roles of dual-specificity phosphatases and their specific characteristics in human cancer. Biochim. Biophys. Acta - Rev. Cancer *1876*, 188562. https://doi.org/10.1016/j.bbcan.2021.188562.
2. Saha, S., Bardelli, A., Buckhaults, P., Velculescu, V.E., Rago, C., St Croix, B., Romans, K.E., Choti, M.A., Lengauer, C., Kinzler, K.W., et al. (2001). A phosphatase associated with metastasis of colorectal cancer. Science *294*, 1343–1346. https://doi.org/10.1126/science.1065817.
3. Bessette, D.C., Qiu, D., and Pallen, C.J. (2008). PRL PTPs: mediators and markers of cancer progression. Cancer Metastasis Rev. *27*, 231–252. https://doi.org/10.1007/s10555-008-9121-3.
4. Miskad, U.A., Semba, S., Kato, H., and Yokozaki, H. (2004). Expression of PRL-3 phosphatase in human gastric carcinomas: close correlation with invasion and metastasis. Pathobiology. *71*, 176–184. https://doi.org/10.1159/000078671.
5. Molleví, D.G., Aytes, A., Padullés, L., Martínez-Iniesta, M., Baixeras, N., Salazar, R., Ramos, E., Figueras, J., Capella, G., and Villanueva, A. (2008). PRL-3 is essentially overexpressed in primary colorectal tumours and associates with tumour aggressiveness. Br. J. Cancer *99*, 1718–1725. https://doi.org/10.1038/sj.bjc.6604747.
6. Sager, J.A., Benvenuti, S., and Bardelli, A. (2004). PRL-3: A phosphatase for metastasis? Cancer Biol. Ther. *3*, 952–953. https://doi.org/10.4161/cbt.3.10.1115.
7. Al-Aidaroos, A.Q.O., Yuen, H.F., Guo, K., Zhang, S.D., Chung, T.H., Chng, W.J., and Zeng, Q. (2013). Metastasis-associated PRL-3 induces EGFR activation and addiction in cancer cells. J. Clin. Invest. *123*, 3459–3471. https://doi.org/10.1172/JCI66824.
8. Stanford, S.M., and Bottini, N. (2023). Targeting protein phosphatases in cancer immunotherapy and autoimmune disorders. Nat. Rev. Drug Discov. *22*, 273–294. https://doi.org/10.1038/s41573-022-00618-w.
9. Zeng, Q., Dong, J.M., Guo, K., Li, J., Tan, H.X., Koh, V., Pallen, C.J., Manser, E., and Hong, W. (2003). PRL-3 and PRL-1 promote cell migration, invasion, and metastasis. Cancer Res. *63*, 2716–2722.
10. Wang, H., Vardy, L.A., Tan, C.P., Loo, J.M., Guo, K., Li, J., Lim, S.G., Zhou, J., Chng, W.J., Ng, S.B., et al. (2010). PCBP1 suppresses the translation of metastasis-associated PRL-3 phosphatase. Cancer Cell *18*, 52–62. https://doi.org/10.1016/j.ccr.2010.04.028.
11. Wang, H., Quah, S.Y., Dong, J.M., Manser, E., Tang, J.P., and Zeng, Q. (2007). PRL-3 down-regulates PTEN expression and signals through PI3K to promote epithelial-mesenchymal transition. Cancer Res. *67*, 2922–2926. https://doi.org/10.1158/0008-5472.CAN-06-3598.
12. Sun, F., Li, W., Wang, L., and Jiao, C. (2017). Expression of phosphatase of regenerating liver-3 is associated with prognosis of Wilms' tumor. Onco. Targets Ther. *10*, 311–317. https://doi.org/10.2147/OTT.S107076.
13. Hong, C.W., and Zeng, Q. (2014). Tapping the treasure of intracellular oncotargets with immunotherapy. FEBS Lett. *588*, 350–355. https://doi.org/10.1016/j.febslet.2013.10.025.

14. Thura, M., Al-Aidaroos, A.Q.O., Yong, W.P., Kono, K., Gupta, A., Lin, Y.B., Mimura, K., Thiery, J.P., Goh, B.C., Tan, P., et al. (2016). PRL3-zumab, a first-in-class humanized antibody for cancer therapy. JCI Insight *1*, e87607. https://doi.org/10.1172/jci.insight.87607.

15. Thura, M., Al-Aidaroos, A.Q., Gupta, A., Chee, C.E., Lee, S.C., Hui, K.M., Li, J., Guan, Y.K., Yong, W.P., So, J., et al. (2019). PRL3-zumab as an immunotherapy to inhibit tumors expressing PRL3 oncoprotein. Nat. Commun. *10*, 2484. https://doi.org/10.1038/s41467-019-10127-x.

16. Thura, M., Ye, Z., Al-Aidaroos, A.Q., Xiong, Q., Ong, J.Y., Gupta, A., Li, J., Guo, K., Ang, K.H., and Zeng, Q. (2021). PRL3 induces polypoid giant cancer cells eliminated by PRL3-zumab to reduce tumor relapse. Commun. Biol. *4*, 923–1011. https://doi.org/10.1038/s42003-021-02449-8.

17. Chee, C.E., Ooi, M., Lee, S.C., Sundar, R., Heong, V., Yong, W.P., Ng, C.H., Wong, A., Lim, J.S.J., Tan, D.S.P., et al. (2023). A Phase I, First-in-Human Study of PRL3-zumab in Advanced, Refractory Solid Tumors and Hematological Malignancies. Target. Oncol. *18*, 391–402. https://doi.org/10.1007/s11523-023-00962-w.

18. Zeng, Q., Hong, W., and Tan, Y.H. (1998). Mouse PRL-2 and PRL-3, two potentially prenylated protein tyrosine phosphatases homologous to PRL-1. Biochem. Biophys. Res. Commun. *244*, 421–427. https://doi.org/10.1006/bbrc.1998.8291.

19. Lin, M.-D., Lee, H.-T., Wang, S.-C., Li, H.-R., Hsien, H.-L., Cheng, K.-W., Chang, Y.-D., Huang, M.-L., Yu, J.-K., and Chen, Y.-H. (2013). Expression of phosphatase of regenerating liver family genes during embryogenesis: an evolutionary developmental analysis among Drosophila, amphioxus, and zebrafish. BMC Dev. Biol. *13*, 18. https://doi.org/10.1186/1471-213x-13-18.

20. Deichmann, C., Link, M., Seyfang, M., Knotz, V., Gradl, D., and Wedlich, D. (2015). Neural crest specification by Prohibitin1 depends on transcriptional regulation of prl3 and vangl1. Genesis *53*, 627–639. https://doi.org/10.1002/dvg.22883.

21. Rivas, D.R., Dela Cerna, M.V.C., Smith, C.N., Sampathi, S., Patty, B.G., Lee, D., and Blackburn, J.S. (2021). A screen of FDA-approved drugs identifies inhibitors of protein tyrosine phosphatase 4A3 (PTP4A3 or PRL-3). Sci. Rep. *11*, 10302–10312. https://doi.org/10.1038/s41598-021-89668-5.

22. Matter, W.F., Estridge, T., Zhang, C., Belagaje, R., Stancato, L., Dixon, J., Johnson, B., Bloem, L., Pickard, T., Donaghue, M., et al. (2001). Role of PRL-3, a human muscle-specific tyrosine phosphatase, in angiotensin-II signaling. Biochem. Biophys. Res. Commun. *283*, 1061–1068. https://doi.org/10.1006/bbrc.2001.4881.

23. Cardoso-Moreira, M., Halbert, J., Valloton, D., Velten, B., Chen, C., Shao, Y., Liechti, A., Ascenção, K., Rummel, C., Ovchinnikova, S., et al. (2019). Gene expression across mammalian organ development. Nature *571*, 505–509. https://doi.org/10.1038/s41586-019-1338-5.

24. Edwards, D.R., Moroz, K., Zhang, H., Mulholland, D., Abdel-Mageed, A.B., and Mondal, D. (2018). PRL-3 increases the aggressive phenotype of prostate cancer cells in vitro and its expression correlates with high-grade prostate tumors in patients. Int. J. Oncol. *52*, 402–412. https://doi.org/10.3892/ijo.2017.4208.

25. Leiphrakpam, P.D., Lazenby, A.J., Smith, L.M., Brattain, M.G., Black, J.D., Wang, J., and Are, C. (2021). Correlation of PRL3 expression with colorectal cancer progression. J. Surg. Oncol. *123*, 42–51. https://doi.org/10.1002/jso.26253.

26. Xing, X., Lian, S., Hu, Y., Li, Z., Zhang, L., Wen, X., Du, H., Jia, Y., Zheng, Z., Meng, L., et al. (2014). Phosphatase of regenerating liver-3 (PRL-3) is associated with metastasis and poor prognosis in gastric carcinoma. J. Transl. Med. *12*, 73. https://doi.org/10.1186/1479-5876-12-73.

27. Yang, Y., Lian, S., Meng, L., Qu, L., and Shou, C. (2017). Antibody Array Revealed PRL-3 Affects Protein Phosphorylation and Cytokine Secretion. PLoS One *12*, e0169665. https://doi.org/10.1371/journal.pone.0169665.

28. Guo, K., Li, J., Wang, H., Osato, M., Tang, J.P., Quah, S.Y., Gan, B.Q., and Zeng, Q. (2006). PRL-3 initiates tumor angiogenesis by recruiting endothelial cells in vitro and in vivo. Cancer Res. *66*, 9625–9635. https://doi.org/10.1158/0008-5472.CAN-06-0726.

29. Hong, X.C., Liang, Q.L., Chen, M., Yang, H.X., Huang, J., Yi, S.L., Wang, Z.W., Liang, H.Y., Zhang, D.Y., and Huang, Z.Y. (2022). PRL-3 and MMP9 Expression and Epithelial-Mesenchymal Transition Markers in Circulating Tumor Cells From Patients With Colorectal Cancer: Potential Value in Clinical Practice. Front. Oncol. *12*, 878639. https://doi.org/10.3389/fonc.2022.878639.

30. Kong, L., Li, Q., Wang, L., Liu, Z., and Sun, T. (2007). The value and correlation between PRL-3 expression and matrix metalloproteinase activity and expression in human gliomas. Neuropathology *27*, 516–521. https://doi.org/10.1111/j.1440-1789.2007.00818.x.

31. Liang, F., Liang, J., Wang, W.Q., Sun, J.P., Udho, E., and Zhang, Z.Y. (2007). PRL3 promotes cell invasion and proliferation by down-regulation of Csk leading to Src activation. J. Biol. Chem. *282*, 5413–5419. https://doi.org/10.1074/jbc.M608940200.

32. Liang, F., Luo, Y., Dong, Y., Walls, C.D., Liang, J., Jiang, H.Y., Sanford, J.R., Wek, R.C., and Zhang, Z.Y. (2008). Translational control of C-terminal Src kinase (Csk) expression by PRL3 phosphatase. J. Biol. Chem. *283*, 10339–10346. https://doi.org/10.1074/jbc.M708285200.

33. Wei, M., Haney, M.G., Rivas, D.R., and Blackburn, J.S. (2020). Protein tyrosine phosphatase 4A3 (PTP4A3/PRL-3) drives migration and progression of T-cell acute lymphoblastic leukemia in vitro and in vivo. Oncogenesis *9*, 6. https://doi.org/10.1038/s41389-020-0192-5.

34. Li, H., Liu, S., Hu, Y., Zhao, B., Sun, Y., and Xu, D. (2020). Endosulfan promotes cell migration via PTP4A3-mediated signaling pathways in HUVECs. Ecotoxicol. Environ. Saf. *192*, 110267. https://doi.org/10.1016/j.ecoenv.2020.110267.

35. Zhang, T., Liu, L., Lai, W., Zeng, Y., Xu, H., Lan, Q., Su, P., and Chu, Z. (2019). Interaction with tumor-associated macrophages promotes PRL-3-induced invasion of colorectal cancer cells via MAPK pathway-induced EMT and NF-κB signaling-induced angiogenesis. Oncol. Rep. *41*, 2790–2802. https://doi.org/10.3892/or.2019.7049.

36. Siegel, D.A., Richardson, L.C., Henley, S.J., Wilson, R.J., Dowling, N.F., Weir, H.K., Tai, E.W., and Buchanan Lunsford, N. (2020). Pediatric cancer mortality and survival in the United States, 2001-2016. Cancer *126*, 4379–4389. https://doi.org/10.1002/cncr.33080.

37. Bhakta, N., Force, L.M., Allemani, C., Atun, R., Bray, F., Coleman, M.P., Steliarova-Foucher, E., Frazier, A.L., Robison, L.L., Rodriguez-Galindo, C., and Fitzmaurice, C. (2019). Childhood cancer burden: a review of global estimates. Lancet Oncol. *20*, e42–e53. https://doi.org/10.1016/S1470-2045(18)30761-7.

38. Registry, S. C. C. *First Report of the Singapore Childhood Cancer Registry 1997-2005*; Singapore Childhood Cancer Registry.

39. Registry, S. C. C. Singapore Childhood Cancer Registry 2003-2007; Singapore Childhood Cancer Registry.

40. Siegel, D.A., King, J., Tai, E., Buchanan, N., Ajani, U.A., and Li, J. (2014). Cancer incidence rates and trends among children and adolescents in the United States, 2001-2009. Pediatrics *134*, e945–e955. https://doi.org/10.1542/peds.2013-3926.

41. Perkins, S.M., Shinohara, E.T., DeWees, T., and Frangoul, H. (2014). Outcome for children with metastatic solid tumors over the last four decades. PLoS One *9*, e100396. https://doi.org/10.1371/journal.pone.0100396.

42. Butler, E., Ludwig, K., Pacenta, H.L., Klesse, L.J., Watt, T.C., and Laetsch, T.W. (2021). Recent progress in the treatment of cancer in children. CA. Cancer J. Clin. *71*, 315–332. https://doi.org/10.3322/caac.21665.

43. Wolfe, J., Orellana, L., Ullrich, C., Cook, E.F., Kang, T.I., Rosenberg, A., Geyer, R., Feudtner, C., and Dussel, V. (2015). Symptoms and Distress in Children With Advanced Cancer: Prospective Patient-Reported Outcomes From the PediQUEST Study. J. Clin. Oncol. *33*, 1928–1935. https://doi.org/10.1200/JCO.2014.59.1222.

44. Suh, E., Stratton, K.L., Leisenring, W.M., Nathan, P.C., Ford, J.S., Freyer, D.R., McNeer, J.L., Stock, W., Stovall, M., Krull, K.R., et al. (2020). Late mortality and chronic health conditions in long-term survivors of early-adolescent and young adult cancers: a retrospective cohort analysis from the Childhood Cancer Survivor Study. Lancet Oncol. *21*, 421–435. https://doi.org/10.1016/S1470-2045(19)30800-9.

45. Parsons, D.W., Janeway, K.A., Patton, D.R., Winter, C.L., Coffey, B., Williams, P.M., Roy-Chowdhuri, S., Tsongalis, G.J., Routbort, M., Ramirez, N.C., et al. (2022). Actionable Tumor Alterations and Treatment Protocol Enrollment of Pediatric and Young Adult Patients With Refractory Cancers in the National Cancer Institute-Children's Oncology Group Pediatric MATCH Trial. J. Clin. Oncol. *40*, 2224–2234. https://doi.org/10.1200/JCO.21.02838.

46. van Tilburg, C.M., Pfaff, E., Pajtler, K.W., Langenberg, K.P.S., Fiesel, P., Jones, B.C., Balasubramanian, G.P., Stark, S., Johann, P.D., Blattner-Johnson, M., et al. (2021). The Pediatric Precision Oncology INFORM Registry: Clinical Outcome and

Benefit for Patients with Very High-Evidence Targets. Cancer Discov. *11*, 2764–2779. https://doi.org/10.1158/2159-8290.CD-21-0094.

47. Mossé, Y.P., Voss, S.D., Lim, M.S., Rolland, D., Minard, C.G., Fox, E., Adamson, P., Wilner, K., Blaney, S.M., and Weigel, B.J. (2017). Targeting ALK With Crizotinib in Pediatric Anaplastic Large Cell Lymphoma and Inflammatory Myofibroblastic Tumor: A Children's Oncology Group Study. J. Clin. Oncol. *35*, 3215–3221. https://doi.org/10.1200/JCO.2017.73.4830.

48. Robinson, G.W., Gajjar, A.J., Gauvain, K.M., Basu, E.M., Macy, M.E., Maese, L.D., Sabnis, A.J., Foster, J.H., Shusterman, S., and Yoon, J. (2019). Phase 1/1B Trial to Assess the Activity of Entrectinib in Children and Adolescents with Recurrent or Refractory Solid Tumors Including Central Nervous System (CNS) Tumors (American Society of Clinical Oncology).

49. Vassal, G., Faivre, L., Geoerger, B., Plantaz, D., Auvrignon, A., Coze, C., Aladjidi, N., Verschuur, A., Icher, C., and Odile, M. (2016). Crizotinib in Children and Adolescents with Advanced ROS1, MET, or ALK-Rearranged Cancer: Results of the AcSe Phase II Trial (American Society of Clinical Oncology).

50. Hargrave, D.R., Bouffet, E., Tabori, U., Broniscer, A., Cohen, K.J., Hansford, J.R., Geoerger, B., Hingorani, P., Dunkel, I.J., Russo, M.W., et al. (2019). Efficacy and Safety of Dabrafenib in Pediatric Patients with. Clin. Cancer Res. *25*, 7303–7311. https://doi.org/10.1158/1078-0432.CCR-19-2177.

51. Foster, J.H., Voss, S.D., Hall, D.C., Minard, C.G., Balis, F.M., Wilner, K., Berg, S.L., Fox, E., Adamson, P.C., Blaney, S.M., et al. (2021). Activity of Crizotinib in Patients with ALK-Aberrant Relapsed/Refractory Neuroblastoma: A Children's Oncology Group Study (ADVL0912). Clin. Cancer Res. *27*, 3543–3548. https://doi.org/10.1158/1078-0432.CCR-20-4224.

52. Ortiz, M.V., Gerdemann, U., Raju, S.G., Henry, D., Smith, S., Rothenberg, S.M., Cox, M.C., Proust, S., Bender, J.G., Frazier, A.L., et al. (2020). Activity of the Highly Specific RET Inhibitor Selpercatinib (LOXO-292) in Pediatric Patients With Tumors Harboring. JCO Precis. Oncol. *4*, 341–347. https://doi.org/10.1200/PO.19.00401.

53. Zhao, X., Kotch, C., Fox, E., Surrey, L.F., Wertheim, G.B., Baloch, Z.W., Lin, F., Pillai, V., Luo, M., Kreiger, P.A., et al. (2021). NTRK Fusions Identified in Pediatric Tumors: The Frequency, Fusion Partners, and Clinical Outcome. JCO Precis. Oncol. *1*, 204–214. https://doi.org/10.1200/PO.20.00250.

54. Mossé, Y.P., Laudenslager, M., Longo, L., Cole, K.A., Wood, A., Attiyeh, E.F., Laquaglia, M.J., Sennett, R., Lynch, J.E., Perri, P., et al. (2008). Identification of ALK as a major familial neuroblastoma predisposition gene. Nature *455*, 930–935. https://doi.org/10.1038/nature07261.

55. George, R.E., Sanda, T., Hanna, M., Fröhling, S., Luther, W., Zhang, J., Ahn, Y., Zhou, W., London, W.B., McGrady, P., et al. (2008). Activating mutations in ALK provide a therapeutic target in neuroblastoma. Nature *455*, 975–978. https://doi.org/10.1038/nature07397.

56. Renfro, L.A., Ji, L., Piao, J., Onar-Thomas, A., Kairalla, J.A., and Alonzo, T.A. (2019). Trial Design Challenges and Approaches for Precision Oncology in Rare Tumors: Experiences of the Children's Oncology Group. JCO Precis. Oncol. *3*, 1–13. https://doi.org/10.1200/PO.19.00060.

57. Berlanga, P., Pierron, G., Lacroix, L., Chicard, M., Adam de Beaumais, T., Marchais, A., Harttrampf, A.C., Iddir, Y., Larive, A., Soriano Fernandez, A., et al. (2022). The European MAPPYACTS Trial: Precision Medicine Program in Pediatric and Adolescent Patients with Recurrent Malignancies. Cancer Discov. *12*, 1266–1281. https://doi.org/10.1158/2159-8290.CD-21-1136.

58. Wong, M., Mayoh, C., Lau, L.M.S., Khuong-Quang, D.A., Pinese, M., Kumar, A., Barahona, P., Wilkie, E.E., Sullivan, P., Bowen-James, R., et al. (2020). Whole genome, transcriptome and methylome profiling enhances actionable target discovery in high-risk pediatric cancer. Nat. Med. *26*, 1742–1753. https://doi.org/10.1038/s41591-020-1072-4.

59. Guo, K., Li, J., Tang, J.P., Tan, C.P.B., Hong, C.W., Al-Aidaroos, A.Q.O., Varghese, L., Huang, C., and Zeng, Q. (2011). Targeting intracellular oncoproteins with antibody therapy or vaccination. Sci. Transl. Med. *3*, 99ra85. https://doi.org/10.1126/scitranslmed.3002296.

60. Yarmarkovich, M., Marshall, Q.F., Warrington, J.M., Premaratne, R., Farrel, A., Groff, D., Li, W., di Marco, M., Runbeck, E., Truong, H., et al. (2021). Cross-HLA targeting of intracellular oncoproteins with peptide-centric CARs. Nature *599*, 477–484. https://doi.org/10.1038/s41586-021-04061-6.

61. Guo, K., Tang, J.P., Jie, L., Al-Aidaroos, A.Q.O., Hong, C.W., Tan, C.P.B., Park, J.E., Varghese, L., Feng, Z., Zhou, J., et al. (2012). Engineering the first chimeric antibody in targeting intracellular PRL-3 oncoprotein for cancer therapy in mice. Oncotarget *3*, 158–171. https://doi.org/10.18632/oncotarget.442.

62. Li, J., Guo, K., Koh, V.W.C., Tang, J.P., Gan, B.Q., Shi, H., Li, H.X., and Zeng, Q. (2005). Generation of PRL-3- and PRL-1-specific monoclonal antibodies as potential diagnostic markers for cancer metastases. Clin. Cancer Res. *11*, 2195–2204. https://doi.org/10.1158/1078-0432.CCR-04-1984.

63. (2022). Peptide-Centric CAR-T Cells Target Unmutated Intracellular Oncoproteins. Cancer Discov. *12*, 12. https://doi.org/10.1158/2159-8290.CD-RW2021-162.

64. Rafiq, S., Purdon, T.J., Daniyan, A.F., Koneru, M., Dao, T., Liu, C., Scheinberg, D.A., and Brentjens, R.J. (2017). Optimized T-cell receptor-mimic chimeric antigen receptor T cells directed toward the intracellular Wilms Tumor 1 antigen. Leukemia *31*, 1788–1797. https://doi.org/10.1038/leu.2016.373.

65. Dao, T., Yan, S., Veomett, N., Pankov, D., Zhou, L., Korontsvit, T., Scott, A., Whitten, J., Maslak, P., Casey, E., et al. (2013). Targeting the intracellular WT1 oncogene product with a therapeutic human antibody. Sci. Transl. Med. *5*, 176ra33. https://doi.org/10.1126/scitranslmed.3005661.

66. Guzińska-Ustymowicz, K., and Pryczynicz, A. (2011). PRL-3, an emerging marker of carcinogenesis, is strongly associated with poor prognosis. Anticancer. Agents Med. Chem. *11*, 99–108. https://doi.org/10.2174/187152011794941145.

67. Ye, Z., Al-Aidaroos, A.Q.O., Park, J.E., Yuen, H.F., Zhang, S.D., Gupta, A., Lin, Y., Shen, H.M., and Zeng, Q. (2015). PRL-3 activates mTORC1 in Cancer Progression. Sci. Rep. *5*, 17046. https://doi.org/10.1038/srep17046.

68. Mu, N., Gu, J., Liu, N., Xue, X., Shu, Z., Zhang, K., Huang, T., Chu, C., Zhang, W., Gong, L., et al. (2018). PRL-3 is a potential glioblastoma prognostic marker and promotes glioblastoma progression by enhancing MMP7 through the ERK and JNK pathways. Theranostics *8*, 1527–1539. https://doi.org/10.7150/thno.22699.

69. Mellström, K., Bjelfman, C., Hammerling, U., and Påhlman, S. (1987). Expression of c-src in cultured human neuroblastoma and small-cell lung carcinoma cell lines correlates with neurocrine differentiation. Mol. Cell. Biol. *7*, 4178–4184. https://doi.org/10.1128/mcb.7.12.4178-4184.1987.

70. Zhang, X., Simerly, C., Hartnett, C., Schatten, G., and Smithgall, T.E. (2014). Src-family tyrosine kinase activities are essential for differentiation of human embryonic stem cells. Stem Cell Res. *13* (3 Pt A), 379–389. https://doi.org/10.1016/j.scr.2014.09.007.

71. Bjelfman, C., Hedborg, F., Johansson, I., Nordenskjöld, M., and Påhlman, S. (1990). Expression of the neuronal form of pp60c-src in neuroblastoma in relation to clinical stage and prognosis. Cancer Res. *50*, 6908–6914.

72. Szklarczyk, D., Gable, A.L., Lyon, D., Junge, A., Wyder, S., Huerta-Cepas, J., Simonovic, M., Doncheva, N.T., Morris, J.H., Bork, P., Jensen, L.J., and Mering, C.V. (2019). STRING v11: protein-protein association networks with increased coverage, supporting functional discovery in genome-wide experimental datasets. Nucleic Acids Res. *47*, D607–D613.

73. Seymour, L., Bogaerts, J., Perrone, A., Ford, R., Schwartz, L.H., Mandrekar, S., Lin, N.U., Litière, S., Dancey, J., Chen, A., et al. (2017). iRECIST: guidelines for response criteria for use in trials testing immunotherapeutics. Lancet Oncol. *18*, e143–e152. https://doi.org/10.1016/S1470-2045(17)30074-8.

OMTO, Volume *30*

Supplemental information

Exploiting frequent and specific expression of PRL3 in pediatric solid tumors for first-in-child use of PRL3-zumab humanized antibody

Amos Hong Pheng Loh, Min Thura, Abhishek Gupta, Sheng Hui Tan, Kelvin Kam Yew Kuan, Koon Hwee Ang, Khurshid Merchant, Kenneth Tou En Chang, Hui Yi Yon, Yong Chen, Mathew Hern Wang Cheng, Arjandas Mahadev, Matthew Chau Hsien Ng, Michaela Su-Fern Seng, Prasad Iyer, Pei Ling Chia, Shui Yen Soh, and Qi Zeng

Table S1. Clinical and pathological features of patients with primary tumors and matched normal tissues

Patient no	Sample ID	Specimen type	Tumor site	Diagnosis	Gender	Age at diagnosis (y)	Extent	PRL3 expr-ession
Neuroblastoma								
1	1T	Primary tumor	Adrenal gland	Nodular ganglioneuroblastoma	Male	3.38	Unknown	+
2	2T	Primary tumor	Retroperitoneum	Differentiating neuroblastoma	Female	3.39	Unknown	+
3	3T	Primary tumor	Retroperitoneum	Differentiating neuroblastoma	Male	2.90	Unknown	+
4	4T	Primary tumor	Retroperitoneum	Intermixed ganglioneuroblastoma	Female	2.67	Metastatic	+
5	5T	Primary tumor	Retroperitoneum	Differentiating neuroblastoma	Male	1.72	Metastatic	+
6	6N	Matched normal	Retroperitoneum	Neuroblastoma	Male	6.22	Unknown	-
7	7T	Primary tumor	Adrenal gland	Neuroblastoma	Male	7.56	Metastatic	+
	7N	Matched normal						-
8	8T	Primary tumor	Liver metastasis	Neuroblastoma	Female	1.74	Metastatic	+
	8N	Matched normal						-
Wilms tumor								
9	1T	Primary tumor	Kidney	Wilms tumor	Male	1.93	Localized	-
10	2T	Primary tumor	Kidney	Wilms tumor	Male	4.28	Localized	-
11	3T	Primary tumor	Kidney	Wilms tumor	Female	0.25	Localized	+
12	4T	Primary tumor	Kidney	Wilms tumor	Female	2.56	Localized	+
	4N	Matched normal						-
13	5T	Primary tumor	Kidney	Wilms tumor	Female	7.22	Localized	-
	5N	Matched normal						-
14	6T	Primary tumor	Kidney	Wilms tumor	Female	2.84	Localized	+
	6N	Matched normal						-
15	7T	Primary tumor	Kidney	Wilms tumor	Female	5.48	Unknown	-
	7N	Matched normal						-
Osteosarcoma								
16	1T	Primary tumor	Tibia	Conventional osteosarcoma	Male	18.35	Localized	-

17	2T	Primary tumor	Femur	Conventional osteosarcoma	Female	12.98	Localized	-
18	3T	Primary tumor	Femur	Conventional osteosarcoma	Male	11.75	Localized	-
19	4T	Primary tumor	Skeletal bone NOS	Conventional osteosarcoma	Male	Unknown	Unknown	+
20	5T	Primary tumor	Lower limb	Conventional osteosarcoma	Female	Unknown	Unknown	+
21	6T	Primary tumor	Lower limb	High grade surface osteosarcoma	Female	Unknown	Unknown	-
22	7M	Metastatic tumor	Lung nodule	Metastatic osteosarcoma	Female	Unknown	Metastatic	+
23	8M	Metastatic tumor	Lung nodule	Metastatic osteosarcoma	Female	Unknown	Metastatic	+
Rhabdomyosarcoma and non-rhabdomyosarcoma soft tissue sarcomas								
24	1T	Primary tumor	Peritoneum	Desmoplastic small round cell tumor	Female	11.16	Regional	+
25	2T	Primary tumor	Lower limb	Alveolar soft part sarcoma	Female	13.22	Regional	+
26	3T	Primary tumor	Chest wall	Undifferentiated sarcoma	Female	13.26	Regional	-
27	4T	Primary tumor	Lower limb	Chondroblastoma	Male	Unknown	Unknown	-
28	5T	Primary tumor	Chest wall	Embryonal rhabdomyosarcoma	Male	3.50	Regional	+
	5N	Matched normal						-
29	6T	Primary tumor	Mandible, parotid	Alveolar rhabdomyosarcoma	Male	1.59	Regional	+
	6N	Matched normal						-
30	7T	Primary tumor	Upper limb and shoulder	Leiomyosarcoma	Male	Unknown	Unknown	+
	7N	Matched normal						-
Renal sarcomas								
31	1T	Primary tumor	Kidney	Rhabdoid tumor of kidney	Male	0.35	Metastatic	+
32	2T	Primary tumor	Kidney	Rhabdoid tumor of kidney	Female	Unknown	Unknown	+
	2N	Matched normal						-
33	3T	Primary tumor	Kidney	Clear cell sarcoma of kidney	Male	Unknown	Unknown	-
	3N	Matched normal						-
34	4T	Primary tumor	Kidney	Clear cell sarcoma of kidney	Male	Unknown	Unknown	+
	4N	Matched normal						-
35	5T	Primary tumor	Kidney	Post-chemotherapy renal sarcoma	Male	Unknown	Unknown	-
	5N	Matched normal						-

Table S2. Clinical and pathological features of patients with primary tumors and matched relapsed tumors

Patient no	Sample ID	Specimen type	Diagnosis	Age at diagnosis (y)	Gender	Extent	Survival status	PRL3 expression
Neuroblastoma								
1	1T	Primary tumor	Differentiating neuroblastoma	6.09	Female	Metastatic	Alive	-
	1R	Relapse tumor		6.39				+
Wilms tumor								
9	1T	Primary tumor	Favorable histology Wilms tumor	5.53	Male	Metastatic	Alive	+
	1R	Relapse tumor		5.91				+
10	2T	Primary tumor	Favorable histology Wilms tumor	4.23	Male	Localized	Dead	+
	2R	Relapse tumor		6.63				+
Osteosarcoma								
16	1T	Primary tumor	High-grade chondroblastic osteosarcoma	8.00	Male	Localized	Dead	+
	1R	Relapse tumor		9.63				-
17	2T	Primary tumor	High-grade conventional osteosarcoma	11.94	Female	Metastatic	Alive	+
	2R	Relapse tumor		14.78				+
18	3T	Primary tumor	High-grade conventional osteosarcoma	8.16	Male	Metastatic	Alive	-
	3R	Relapse tumor		9.14				+
Rhabdomyosarcoma and non-rhabdomyosarcoma soft tissue sarcoma								
24	1T	Primary tumor	Malignant mesothelioma	14.35	Female	Metastatic	Alive	-
	1R	Relapse tumor		14.55				-
25	2T	Primary tumor	PAX7-FKHR translocation positive alveolar rhabdomyosarcoma	2.33	Male	Metastatic	Alive	-
	2R	Relapse tumor*		5.18				+

*Tumor from second relapse; all other relapse tumors listed above are from first relapse episode

Table S3: Treatment history of initial tumor & serial relapses

Type of Treatment	Treatment regimen	Duration
Initial treatment		
1. Chemotherapy	IVADO (ifosfamide, vincristine, actinomycin, doxorubicin) according to EpSSG RMS 2005 metastatic arm	6 months
2. Surgery	Local control resection with regional lymph node sampling	1 day
3. Radiotherapy	Resected primary tumor site and regional nodal basin	1 month
4. Maintenance Chemotherapy	Oral cyclophosphamide and IV vinorelbine	12 months
First relapse (2 months after stopping maintenance therapy)		
1. Salvage chemotherapy	VIT (vincristine, irinotecan, temozolomide)	8 cycles over 6 months
2. Radiotherapy	Whole lung with boost to involved areas	6 weeks
Second relapse (7 months after radiotherapy)		
1. Short bridging chemotherapy	IV vinorelbine and oral cyclophosphamide	10 days
2. Salvage chemotherapy	VTC (vincristine, topotecan, cyclophosphamide)	7 cycles over 5 months
3. Surgery	Local control resection of distant relapse sites	1 day
4. Radiotherapy	Resected distant relapse sites	6 weeks
5. Experimental NK cell therapy	Activated NK cell infusion	1 day
Third relapse (1 month after activated NK cell infusion)		
1. Salvage chemotherapy	IV bevacizumab, gemcitabine, docetaxel	8 cycles over 6 months
2. Surgery	Drainage of persistent malignant effusion	
3. Maintenance therapy	IV temsirolimus and vinblastine	1 month
	IV vinblastine	2 months
Fourth relapse (during maintenance therapy)		1 day
1. Palliative chemotherapy	IV carboplatin and etoposide	8 cycles over 6 months
2. Palliative radiation	Focal to involved distant sites	2 sessions
Fifth relapse (1 month after IV carboplatin & etoposide)		

EpSSG: European pediatric Soft Tissue Sarcoma Study Group; RMS: rhabdomyosarcoma; IV: intravenous; NK: Natural killer cells

Table S4: Salvage strategy treatment plan for 5th relapse

No.	Type of Treatment	Days	Treatment regimen
1	Bridging salvage chemo	-21	IV Vinorelbine dose 1
		-14	IV Vinorelbine dose 2
2	Hypofractionated palliative RT to left chest tumors	-11	Session 1
		-10	Session 2
3	PRL3-zumab compassionate trial	1*	C1D1 4mg/kg in 2 divided doses
		8	C1D8 (4mg/kg)
		18	C2D1 (4.8mg/kg)
4	Hypofractionated palliative RT to left chest tumors	22	Session 3
		23	Session 4
5	PRL3-zumab compassionate trial	28	C2D11 (5.5mg/kg)
	Disease assessment	37	CT chest / Whole Body PET-CT
6	PRL3-zumab compassionate trial	38	C3D1 (5mg/kg)
		48	C3D11 (5mg/kg)

IV: intravenous, CT: computed tomography scan

*C1D1 considered as day 1

Table S5: PRL3-zumab treatment record

Cycle	Day	Body weight	Actual Dose (mg)	PK sample collection
1	1 (C1D1)	14.2 kg	28mg (2mg/kg, AM)<br>28mg (2mg/kg, PM)	pre-C1D1 dose 1 & end of infusion<br>pre-C1D1 dose 2 & end of infusion
	8 (C1D8)	14.3 kg	57mg (4mg/kg)	pre-C1D8 & end of infusion
2	1 (C2D1)	14.7 kg	70mg (4.8 mg/kg)	pre-C2D1 & end of infusion
	11(C2D11)		80mg (5.4mg/kg)	

Table S6: Blood parameters at treatment time points

Parameter	Range	Baseline (day -27)	Baseline (day -23)	Pre-chemo (day -20)	Pre-chemo (day -13)	After RT (day -6)	Pre-C1D1 (day 1)*	Pre-C1D8 (day +8)	Pre-C2D1 (day +18)
Full Blood Count									
Haemoglobin	13.5 – 18 g/L	11.2	10.6	9.5	8.5	7	11	11	11.6
WBC count	4.0 – 11.0 x10^9/L	4.4	5.49	4.41	2.02	0.63	7.38	4.73	3.86
Platelet count	140 – 440 x 10^9/L	45	27	32	33	54	48	60	68
RBC count	4.5 – 6.5 x10^9/L	3.82	3.73	3.25	2.9	2.39	3.82	3.71	3.7
Lymphocyte	1.5 – 4 x10^9/L	1.19	1.15	0.68	0.53	some	0.59	0.54	0.62
Neutrophil	2 – 7.5 x 10^9/L	2.68	3.68	2.89	1.37	few	5.46	3.43	2.2
Monocyte	0.2 – 0.8 10^9/L	0.48	0.6	0.82	0.12		1.25	0.69	0.85
Eosinophil	0.04 – 0.4 x 10^9/L	0.04	0.05	0	0		0	0.02	0.15
Basophil	<0.21 x10^9/L	0	0	0.02	0		0	0.05	0.04
Liver Function Test									
Total protein	60-80 g/L	55		55		58	62		
Albumin	40-51 g/L	33	33	35	36	36	40	40	
Total bilirubin	1.7 -20.5 umol/L	8		4		4	6		
Direct bilirubin	<5.1 umol/L	4		<2		<2	3		
ALP	<130 U/L	190		224		208	204		
ALT	<51 U/L	24		31		34	48		
AST	<51 U/L	32		40		39	44		

WBC: white blood cell, RBC: red blood cell, ALP: alkaline phosphatase, ALT: alanine transaminase, AST: aspartate transaminase
*C1D1 considered as day 1

Fig S1

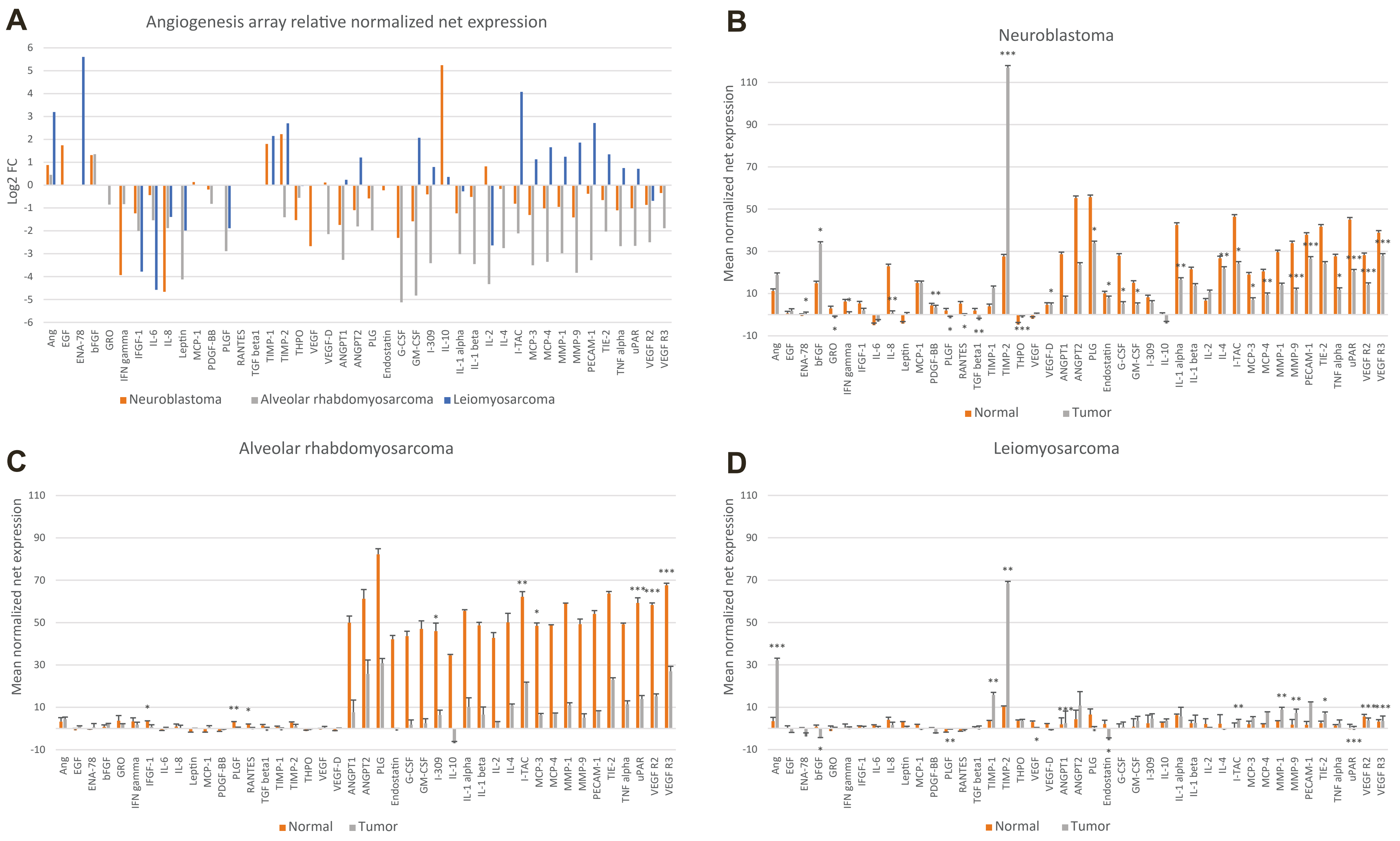

Fig S1. PRL3 expression is associated with elevation of pro angiogenesis factors. (A) Paired bar graph showing log2 fold-change of semi-quantitative intensity of Western blot signal of pro-angiogenic factors detected in pediatric tumor samples relative to matched adjacent normal tissue. Paired bar graphs of mean normalized semi-quantitative intensity of Western blot signal of pro-angiogenic factors in neuroblastoma (B), alveolar rhabdomyosarcoma (C) and leiomyosarcoma (D), with corresponding matched adjacent normal tissues.
